# Providing Equitable Care for Patients With Non-English Language Preference in Telemedicine: Training on Working With Interpreters in Telehealth

**DOI:** 10.15766/mep_2374-8265.11367

**Published:** 2023-12-14

**Authors:** Tiffany M. Shin, Kristen A. Dodenhoff, Mariana Pardy, Abigail Smith Wehner, Samuel Rafla, Leslie Doroski McDowell, Nancy M. Denizard-Thompson

**Affiliations:** 1 Assistant Professor, Department of Pediatrics, Wake Forest University School of Medicine; 2 Second-Year Resident, Department of Family and Community Medicine, Wake Forest University School of Medicine; 3 Project Manager, Department of Social Sciences and Health Policy, Wake Forest University; 4 Third-Year Resident, Department of Emergency Medicine, Wake Forest University School of Medicine; 5 Third-Year Resident, Department of Anesthesiology, Icahn School of Medicine at Mount Sinai; 6 Quality Improvement Specialist and Curriculum Developer, Northwest Area Health Education Center; 7 Associate Professor, Department of Internal Medicine, Wake Forest University School of Medicine

**Keywords:** Interpreter, Non-English Speaking, Clinical/Procedural Skills Training, Online/Distance Learning, Diversity, Equity, Inclusion, Language-Appropriate Health Care, Telehealth

## Abstract

**Introduction:**

The COVID-19 pandemic has led to a large increase in telemedicine encounters. Despite this rise in virtual visits, patients who speak non-English languages have experienced challenges accessing telemedicine. To improve health equity, medical education on telehealth delivery should include instruction on working with interpreters in telehealth.

**Methods:**

We developed a 25-minute self-directed module with collective expertise of faculty with experience in medical education, interpreter training, and communication training. The module was delivered online as part of a longitudinal health equity curriculum for third-year medical students. In addition to didactic information, the module contained video examples of interpreter interactions in telehealth.

**Results:**

Sixty-four third-year medical students participated in the study, and 60 completed a postmodule survey. Students were satisfied with the content of the module, as well as the duration of time required to complete the tasks. Approximately 90% would recommend it to future students. Nearly 80% of students rated the module as being quite effective or extremely effective at increasing their comfort level with visits with patients with non-English language preference.

**Discussion:**

Our module provides a basic framework for medical students on how to successfully work with interpreters during a language-discordant virtual visit. This format of asynchronous learning could also be easily expanded to resident physicians and faculty seeking more resources around working with interpreters in telemedicine.

## Educational Objectives

By the end of this session, participants will be able to:

1. Summarize how to access interpreters in the telehealth setting.
2. Recognize best practices when working with interpreters in the telehealth setting.
3. Build confidence in their skill to engage patients with non-English preference in telehealth settings.

## Introduction

The COVID-19 pandemic resulted in a dramatic increase in telemedicine encounters.^1^ Despite this rise in virtual visits, patients who speak non-English languages have experienced challenges accessing telemedicine.^2^ Sixty-one million people in the U.S. report speaking a non-English language at home, most commonly Spanish and Chinese, and four out of 10 Hispanic individuals in the U.S. report non-English language preference (NELP).^3,4^

Patients with NELP are less likely to be insured, less likely to receive timely and regular primary care, and less likely to adhere to recommendations provided by physicians.^5^ One study found that patients with NELP are more likely to have prolonged hospital stays when compared to patients for whom English is their primary language.^6^

Research has highlighted the benefits of working with interpreters during language-discordant clinical encounters, including decreased readmission rates,^7^ increased health care utilization, and improved patient outcomes.^8^ However, there are many barriers that prevent the effective use of interpreters by clinicians. Clinicians frequently use ad hoc interpreters (e.g., family, untrained staff) who commit errors with clinical significance at twice the rate of a professional interpreter.^9^ Despite a recognized need, clinicians underutilize professional medical interpreters. In addition to their training in faithful, accurate, and direct interpretation of complex medical information, professional interpreters are often able to provide additional cultural insights to providers and smooth over communication difficulties that may arise due to cultural differences.^10^

Of all the factors identified as challenges, the lack of clinician knowledge on how to work with interpreters has been found to be most significant.^11^ One study found that 78% of clinicians reported occasional to frequent troubles integrating interpreters into the telemedicine encounter.^12^ Given that telemedicine will likely remain a cornerstone of patient care after the COVID-19 pandemic, physicians-in-training, both residents and medical students, must be able to effectively incorporate interpreters into these patient encounters. By doing this, they can better provide equitable telemedicine care for patients with NELP. A PubMed search of existing research on interpreter use in telemedicine shows that there are limited studies on this topic—in fact, patients with NELP were noted to be less likely to complete telehealth visits.^13^ Similarly, a review of *MedEdPORTAL* publications shows several modules on cultural competency when working with patients with NELP and the benefit of utilizing simulation to help providers become more comfortable with utilizing in person interpreters, but there do not appear to be any modules specifically on working with interpreters in a telehealth setting.^14,15^

To address this lack of existing training, our team designed an asynchronous module teaching learners how to effectively work with an interpreter during a telehealth encounter, including how to access interpreters in a variety of telehealth modalities, proper camera positioning, communication techniques, dealing with cultural differences, and more. The module was created using the collaborative expertise and experiences of the team members in medical education, clinical communication skills training, and interpreter training. We used Kern's six steps for curriculum design to develop our module.^16^ We did an informal needs assessment of faculty and learners in the ambulatory setting to determine that there was a need for education on effective use of interpreters for telehealth. We were not able to identify guidelines for best practices in telehealth for patients with NELP; however, we used the National Standards of Practice for Interpreters in Health Care, adapting them to a telemedicine setting.^17^ Regarding standards for telehealth, we followed guidance from the Academy of Communication in Healthcare in preparing the content.^18^ An asynchronous online model was chosen to allow for self-paced learning and easy dissemination of the module, as the goal was to share this module widely.

## Methods

We developed a self-directed module intended to teach medical students how to effectively work with interpreters during a telemedicine visit with patients with NELP. There was limited education in telehealth in the preclinical years for students at our institution, and most of the teaching on telemedicine and interpreter use typically happened during actual clinical encounters, which presented a challenge for learners and providers alike. We delivered the module during the third year as this was when students would be engaging with patients in the ambulatory setting. In addition to general themes, we wanted to share practical information about setup and logistics that would be best served if delivered when students were actively participating in patient care. A prerequisite was not required prior to completing the module; however, our students did have a session on working with interpreters in their preclinical curriculum. We created the training as an asynchronous module to offer flexibility and schedule convenience and to allow learners to work at their own pace. In addition, we wanted to create something that students could access easily if they needed a refresher or wanted to revisit the material.

The module was developed to be integrated into the third-year medical students’ transition to patient care orientation prior to starting clinical rotations; however, given the amount of content during this curriculum, it could only be offered as a voluntary module. Therefore, because of concerns that students might not complete the module if it was voluntary, we also embedded it in the pediatric clerkship in the Longitudinal Health Equity Curriculum,^19^ where it was a mandatory assignment. The Longitudinal Health Equity Curriculum was a thread running through the third-year clerkships and focusing on social determinants of health and equity in health care. Each third-year clerkship had a focus and theme. The theme during the pediatric clerkship was care of patients with NELP, and this was also one of the clerkships where students had the greatest number of ambulatory encounters and opportunities to practice the content of the module. During the third year, there were approximately 10 students per 6-week block on the pediatric rotation. The students could complete the module anytime during the 6-week interval.

The module included interactive slides and two videos that we developed and recorded. No actual patients were involved in the development of the recordings or other learning materials.

The intended audience for the module was third-year medical students; however, the module would be appropriate for all levels of medical students, residents, fellows, advanced practice providers, and physicians providing care for patients with NELP via telemedicine.

An assignment was created in Canvas, our learning system, with a link and instructions on completion. Students had to enter a comment or screenshot on the assignment that had been completed or was completed during the transition to patient care orientation.

Instructions on how to use the module are included in Appendix A. Created using Articulate software, this interactive module reviews best practices in telehealth when using an interpreter. The module cannot be edited; however, we recommend providing supplemental information to learners about specifics of accessing telehealth at one's institution. This could be done as a handout with screenshots and how-to information. The module highlights how to properly position oneself for a visit. It reviews basic introductions, consent, screening for language preference, and privacy. Additionally, it reviews best practices for physicians and things to look for in an interpreter. It also shares important cultural considerations that are unique to telehealth. The module is provided in Appendix B as an Articulate zip file.

### Options for an In-Person Version

Although not the version that we implemented, for the sake of benefiting from the component elements we have provided some additional resources in case faculty are interested in a faculty-facilitated version of the module. The resources are also offered to allow for customizability. Appendix C features a facilitator guide and gives guidance on how to use the PowerPoint with some small-group breakouts for an interactive session if opting not to use the Articulate module. The videos are incorporated into the module; however, we also present them as Appendices D and E for use within the module or as separate educational materials. Appendix D highlights potential pitfalls, such as not speaking directly to the patient, use of medical jargon, and distractions. Appendix E highlights things that promote a good visit, such as clear introductions, minimized distractions, and thanking the interpreter. We have also included Appendix F, the PowerPoint slide version of the module referenced in Appendix B, if one would like to create a didactic session. Appendix G contains a handout that can be either given out to learners as a resource or made into a pocket card. It summarizes the key steps and best practices for a telehealth visit using an interpreter. Appendix H contains a postsurvey to provide feedback on the session's effectiveness. This survey is a standard one used in our medical school to evaluate curricula, and we have adjusted the questions to assess our module's content. In our implementation, the postmodule evaluation was distributed as a link to an online survey at the end of the module. The survey can be delivered using any type of electronic database, or a paper copy can be provided to participants.

## Results

The module was delivered to 64 third-year medical students, of whom 60 completed a postmodule evaluation, for a response rate of 94%. The module was delivered voluntarily as part of a transitions to patient care orientation and as a mandatory assignment as part of a longitudinal curriculum during the pediatric clerkship. Only four students completed the assignment when it was voluntary during the transitions to patient care week. The remaining students completed it during the 6 weeks of the clerkship. The length of time required to complete the module was approximately 25 minutes. This was very satisfactory for all the participants, with 98% indicating that they felt the amount of time required to complete the module was reasonable.

**Figure 1.**
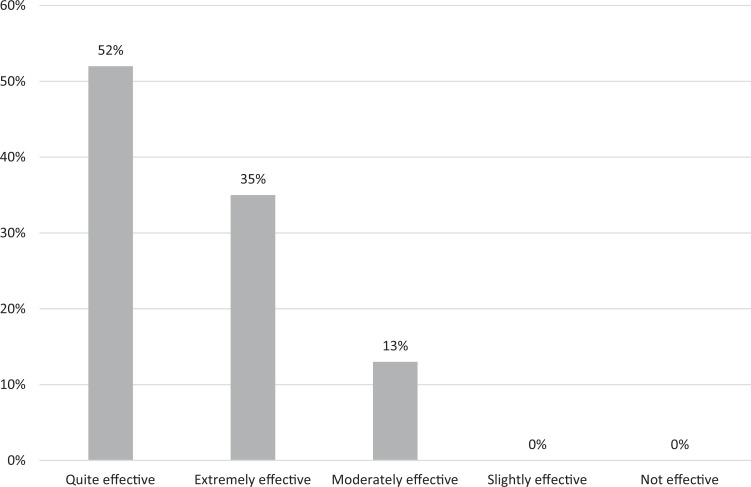
Students’ evaluation of the online module's effectiveness regarding how to work with interpreters (*N* = 60).

**Figure 2.**
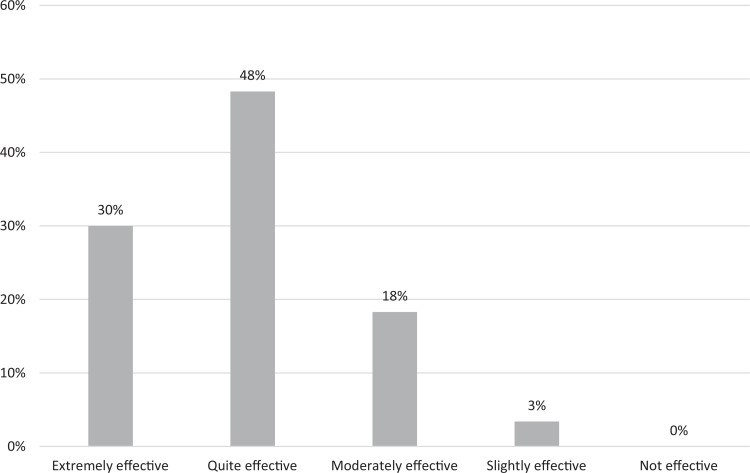
Students’ evaluation of the online module's effectiveness in increasing their comfort with patients with non-English language preference (*N* = 60).

Regarding the separate lessons, 50% of students rated the segment on the basics of accessing interpreters, which went over specifics of accessing telehealth at our institution, as quite effective, and 28% rated it as extremely effective. Twenty-one percent of students rated that lesson as being moderately effective. A similar breakdown was seen for the lesson on how to effectively work with interpreters, with 52% rating it as quite effective, 35% rating it as extremely effective, and 13% rating it as moderately effective (Figure 1). When assessing the lesson on increasing comfort level with visits with patients with NELP, 30% rated it as extremely effective, 48% as quite effective, and 18% as moderately effective (Figure 2).

Student satisfaction with the module was high, with 88% of students indicating that they would recommend this lesson to other students and 4% saying they would recommend it if some changes were made to the module. We were not able to discern any discrete changes. In retrospect, we should have put a comment field in this section so that students could specifically share what they would recommend be modified. Three of the 60 students said they would not recommend the module.

Qualitative feedback was also collected. Several students appreciated that the module was not extremely time intensive and that they had the option to watch videos at their own pace. Other students noted that they would have enjoyed having an in-person component to the training as well as the opportunity to observe a telehealth visit prior to performing one on their own. Two students wanted more explicit instructions on how to access interpreters prior to the visit.

Overall, the module was well received, with 14 students providing direct positive feedback on the content and quality of the modules, including the comments listed below.

• “Manageable, helpful, straight forward.”
• “Concise and informative.”
• “Thank you! Clear module.”
• “Very helpful and time efficient.”
• “I thought this module was succinct but effective and covered all very important points.”
• “I learn best from viewing examples.”

We received two comments providing negative feedback or suggestions for improvement.

• “I don't personally prefer this method if learning, I prefer in person discussion.”
• “In the nuts-and-bolts section, it could be useful to add a video showing someone accessing the video visit/interpreter in the patient chart—this would improve familiarity.”

## Discussion

Our module addresses an important gap in existing training on working with interpreters during telehealth visits. This has become an important skill for providers due to the increase in telehealth since the COVID-19 pandemic. The module provides a basic framework for medical students on how to successfully work with interpreters during a language-discordant virtual visit. Another strength is that the module is delivered asynchronously, which allows for self-paced learning and flexibility in scheduling. During COVID-19, many medical schools had to transition to online learning, and a recent study has shown that students engaged very well with this format, without significant differences in final academic performance.^20^ Another study looked at integrating a pathology thread into the third-year clerkships. The authors found that this asynchronous education component provided a flexible method to reinforce concepts and became a well-received adjunct to the medical education curriculum.^21^

Overall, the module was effective in meeting the educational objectives, and students seemed satisfied with its content and the duration of time required to complete the tasks. Nearly 80% of students rated the module as being quite effective or extremely effective at increasing their comfort level with visits with patients with NELP. It will be difficult to fully assess how successfully we addressed comfort level with working with interpreters in the absence of further assessments. Overall, given the high level of satisfaction with the module, students may be more likely to take the lessons they learned into their clinical practice.

In evaluating the student feedback, we found a desire for more training in telemedicine and in working with interpreters. It may be beneficial to tie this online training to an in-person session on working with interpreters. This could allow students to learn about strategies interpreters use in different settings and to practice unique skills that come with each type of patient encounter. Increasing clinician comfort with in-person interpreters will translate to greater comfort in the virtual setting as well. This is essential to ensuring that we are delivering equitable care to all patients, regardless of language preference.

The module was implemented in a longitudinal curriculum, which meant students were accessing the information at different points of the year. It may be beneficial to embed the module at the beginning of the year, so that all students have access prior to starting clinical rotations. Thus, we plan to work with the course director to see if, given the positive feedback of learners, this resourcecould be included as a mandatory module earlier in the year. General concepts of interpreter and telemedicine use could also be discussed in the preclinical years as well.

One limitation of delivering the module asynchronously versus in person is that students have less interaction with a faculty member and cannot ask direct questions of the facilitator to clarify concepts. However, in the future, we will consider adding a question box to the module so that students can send questions and create an open dialogue. Another limitation to our module is that we did not administer a premodule survey. This makes it more difficult to assess student perceptions of working with an interpreter via telehealth and the module's direct impact. Additional limitations include that the module was administered only to medical students, who often have more limited telehealth experiences in general. The module could be very easily distributed to resident physicians, advanced practice providers, and faculty with little to no adjustment of the content. This would make survey data more generalizable. The module will be available from Wake Forest School of Medicine for all to use. We feel that this is a reasonable future step for this resource and that the module could be a valuable tool for all trainees and practicing physicians.

## Appendices


Module Instructions.docxEquitable Care in Telemedicine folderFacilitator Guide for Alternative Teaching Options.docxInterpreter Room for Improvement Example.mp4Interpreter Better Example.mp4Working With Interpreters in Telehealth.pptxTips for Best Practices With Interpreters Handout.docxPostsurvey.docx
*All appendices are peer reviewed as integral parts of the Original Publication.*
