# Supplementary material for: Providing Equitable Care for Patients With Non-English Language Preference in Telemedicine: Training on Working With Interpreters in Telehealth
Source: MedEdPORTAL. 2023 Dec 14;19:11367. doi: 10.15766/mep_2374-8265.11367 (PMC10719426; doi:10.15766/mep_2374-8265.11367)

Appendix A: Working With Interpreters in Telehealth

Instructions for Facilitators:

The module will take learners approximately 25 minutes to complete. The module cannot be edited. However, we would recommend that you consider providing learners with supplemental information on how to access your institution’s telehealth platform in the form of a how to guide or screen shots.

In addition, we would recommend attaching a copy of Appendix G – Tips for Best Practices With Interpreters to the assignment as a take home handout.

An evaluation link is not included in the module so you may consider creating an evaluation link using the survey provided in Appendix H.

The module is provided to you as an Articulate Module Appendix B entitled Equitable Care in Telemedicine.


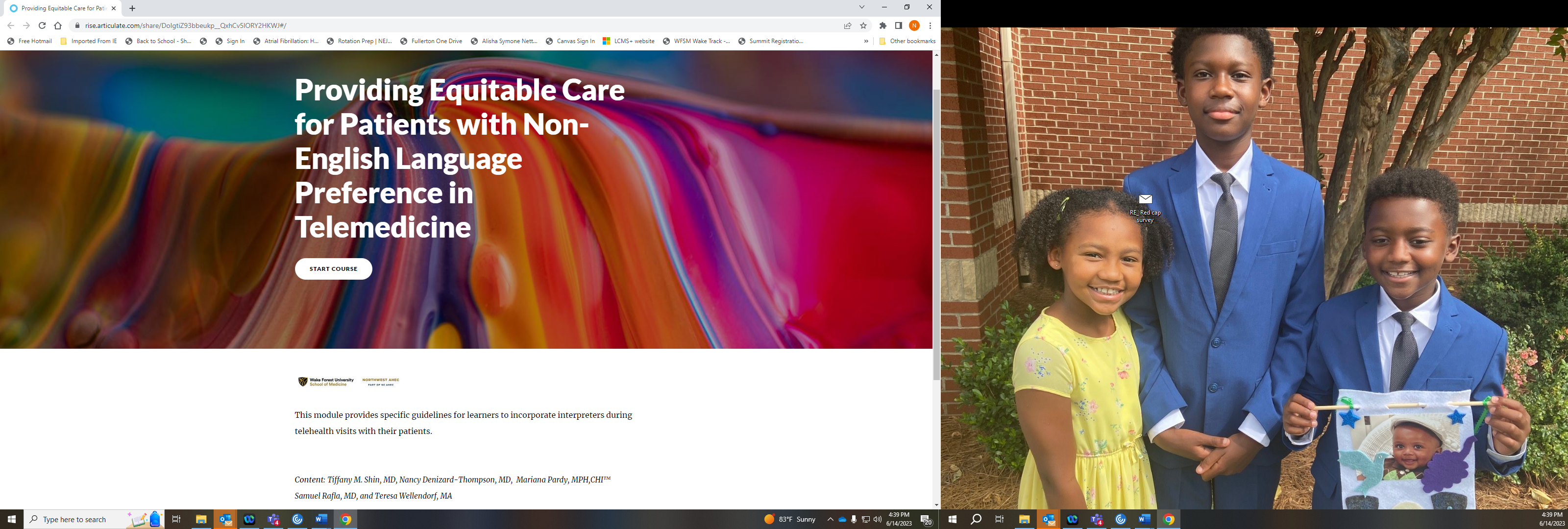

Supplement: Supplementary file 1 — Module Instructions.docxEquitable Care in Telemedicine folderFacilitator Guide for Alternative Teaching Options.docxInterpreter Room for Improvement Example.mp4Interpreter Better Example.mp4Working With Interpreters in Telehealth.pptxTips for Best Practices With Interpreters Handout.docxPostsurvey.docx [file mep_2374-8265.11367-s001.zip › A. Module Instructions.docx]
