# Supplementary material for: Providing Equitable Care for Patients With Non-English Language Preference in Telemedicine: Training on Working With Interpreters in Telehealth
Source: MedEdPORTAL. 2023 Dec 14;19:11367. doi: 10.15766/mep_2374-8265.11367 (PMC10719426; doi:10.15766/mep_2374-8265.11367)
Supplement: Supplementary file 1 — Module Instructions.docxEquitable Care in Telemedicine folderFacilitator Guide for Alternative Teaching Options.docxInterpreter Room for Improvement Example.mp4Interpreter Better Example.mp4Working With Interpreters in Telehealth.pptxTips for Best Practices With Interpreters Handout.docxPostsurvey.docx [file mep_2374-8265.11367-s001.zip › B. Equitable Care in Telemedicine/content/goodbye.html]

Goodbye


👋 Bye!

You may now leave this page.
