# Supplementary material for: Providing Equitable Care for Patients With Non-English Language Preference in Telemedicine: Training on Working With Interpreters in Telehealth
Source: MedEdPORTAL. 2023 Dec 14;19:11367. doi: 10.15766/mep_2374-8265.11367 (PMC10719426; doi:10.15766/mep_2374-8265.11367)
Supplement: Supplementary file 1 — Module Instructions.docxEquitable Care in Telemedicine folderFacilitator Guide for Alternative Teaching Options.docxInterpreter Room for Improvement Example.mp4Interpreter Better Example.mp4Working With Interpreters in Telehealth.pptxTips for Best Practices With Interpreters Handout.docxPostsurvey.docx [file mep_2374-8265.11367-s001.zip › C. Facilitator Guide for Alternative Teaching Options.docx]

**Appendix C: Facilitator Guide for Alternative Teaching Options**

**Time Needed**: Approximately 60 minutes

**Materials Needed:**

- PowerPoint Presentation
- Group of Eager Learners
- Instructor
- Handout with small group questions
- Optional- Invite a guest from language services to share additional tips, strategies, or policies for telehealth or in person interpreter services in your medical center
- Appendix G: Tips for Best Practices With Interpreters- printed on paper or printed into a pocket card

**Directions for Facilitator:**  If you are interested in creating an in-person session rather than using the module located in Appendix B this is an alternative teaching option. This session could be delivered as a didactic session with some interactive breakout sessions. If available, you may consider involving someone from language services at your institution. For this session we will focus on a timeline to just focus on Interpreters in Telehealth. However, you could consider doing a combined session where you talk about use of Interpreters in both telehealth and face to face clinical encounters. In addition, if you are doing a session on best practices in telehealth you could incorporate some of this information into the talk.

Prior to the session would recommend reviewing the slides and updating slide 3 with screenshots or information about your institution’s telehealth platforms.

The video examples in slides 10 and 13 could be viewed during the talk and then the handout that follows could be used to create small breakout groups or self-reflection.

Proposed timeline

1. Introduction- Slides 1-9- (10 minutes)
2. Example A- video (5:30 minutes)
3. Small Group Break out (8 minutes)
4. Large Group discussion/debrief- slide 12- (5 minutes)
5. Example B- video (10 minutes)
6. Small Group (8 minutes)
7. Large Group discussion/debrief- slide 15 (5 minutes)
8. Conclusion- slides 16 and 17-(5 minutes) pass out Appendix G

Small Group Break Out sessions

You will work with a partner or small group to discuss the following as you are viewing the video clip.

Example A- Room for Improvement

**Part I: In the following clip, write down at least 3 things that you notice the medical student does or doesn’t do that impedes a successful patient encounter.**

1.

2.

3.

4.

5.

**Part II: What are your observations of the interaction?**
-Things that worked well

-Things that could be improved on

Small Group Break Out sessions

You will work with a partner or small group to discuss the following as you are viewing the video clip.

Example B- The Better Example

**Part I: In the following clip, write down at least 3 things that you notice the medical student does or doesn’t do that promotes a successful patient encounter.**

1.

2.

3.

4.

5.

**Part II: What are your observations of the interaction?**
-Things that worked well

-Things that could be improved on
