# Supplementary material for: Providing Equitable Care for Patients With Non-English Language Preference in Telemedicine: Training on Working With Interpreters in Telehealth
Source: MedEdPORTAL. 2023 Dec 14;19:11367. doi: 10.15766/mep_2374-8265.11367 (PMC10719426; doi:10.15766/mep_2374-8265.11367)
Supplement: Supplementary file 1 — Module Instructions.docxEquitable Care in Telemedicine folderFacilitator Guide for Alternative Teaching Options.docxInterpreter Room for Improvement Example.mp4Interpreter Better Example.mp4Working With Interpreters in Telehealth.pptxTips for Best Practices With Interpreters Handout.docxPostsurvey.docx [file mep_2374-8265.11367-s001.zip › F. Working With Interpreters in Telehealth.pptx]

## Slide 1
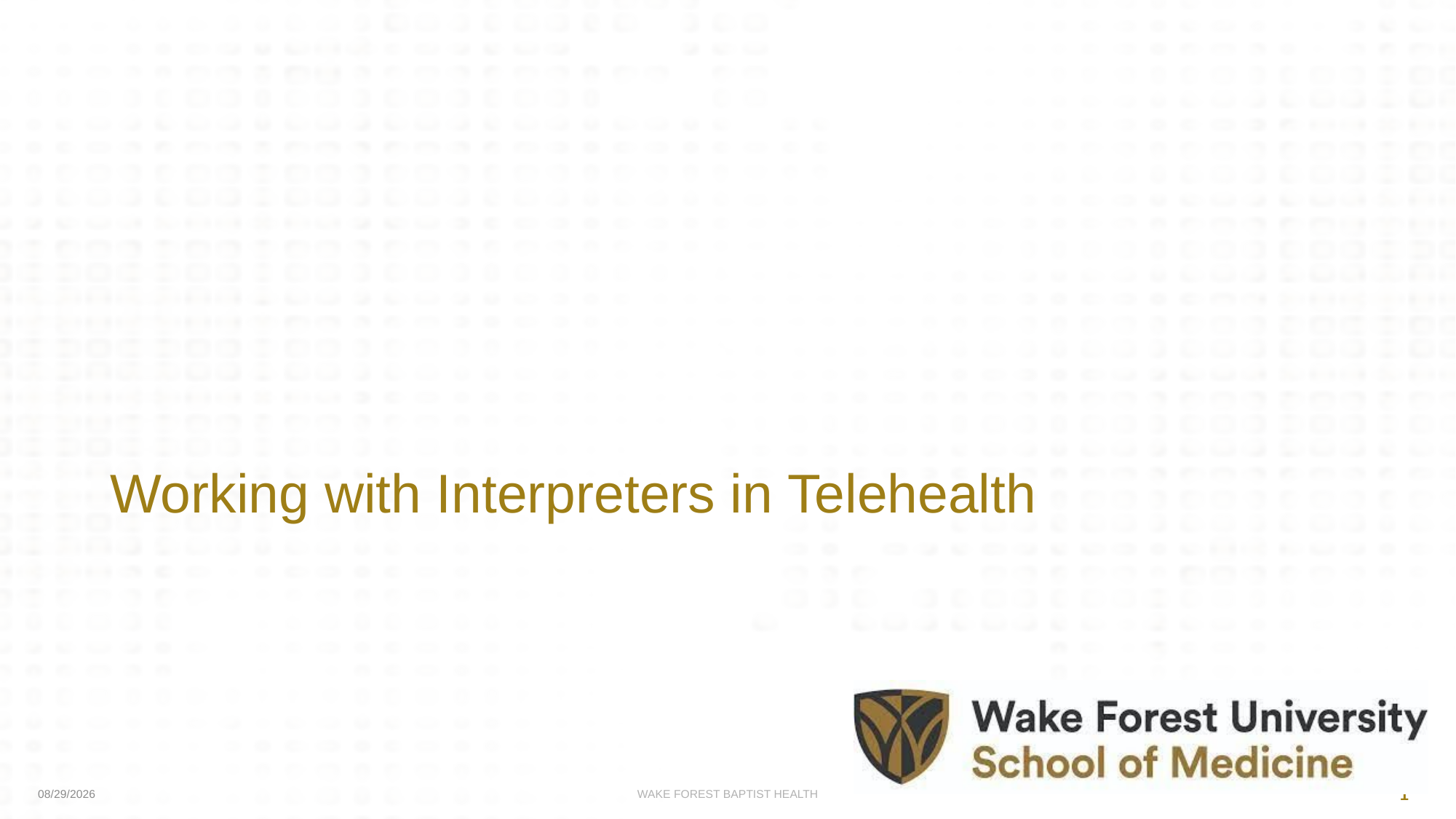

# Working with Interpreters in Telehealth
6/16/2023
WAKE FOREST BAPTIST HEALTH
1

## Slide 2
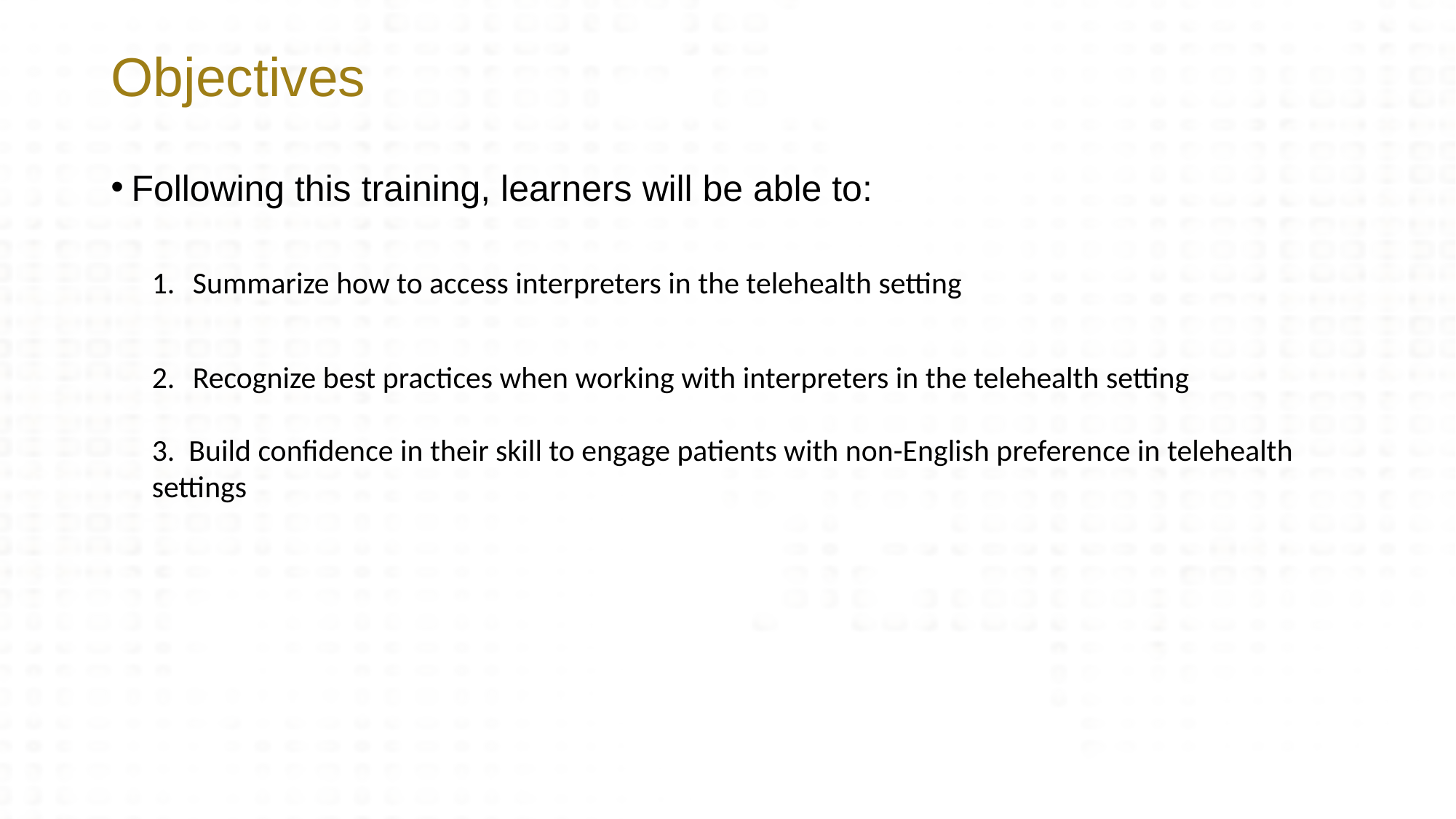

# Objectives
Following this training, learners will be able to:
Summarize how to access interpreters in the telehealth setting
Recognize best practices when working with interpreters in the telehealth setting
3. Build confidence in their skill to engage patients with non-English preference in telehealth settings

## Slide 3
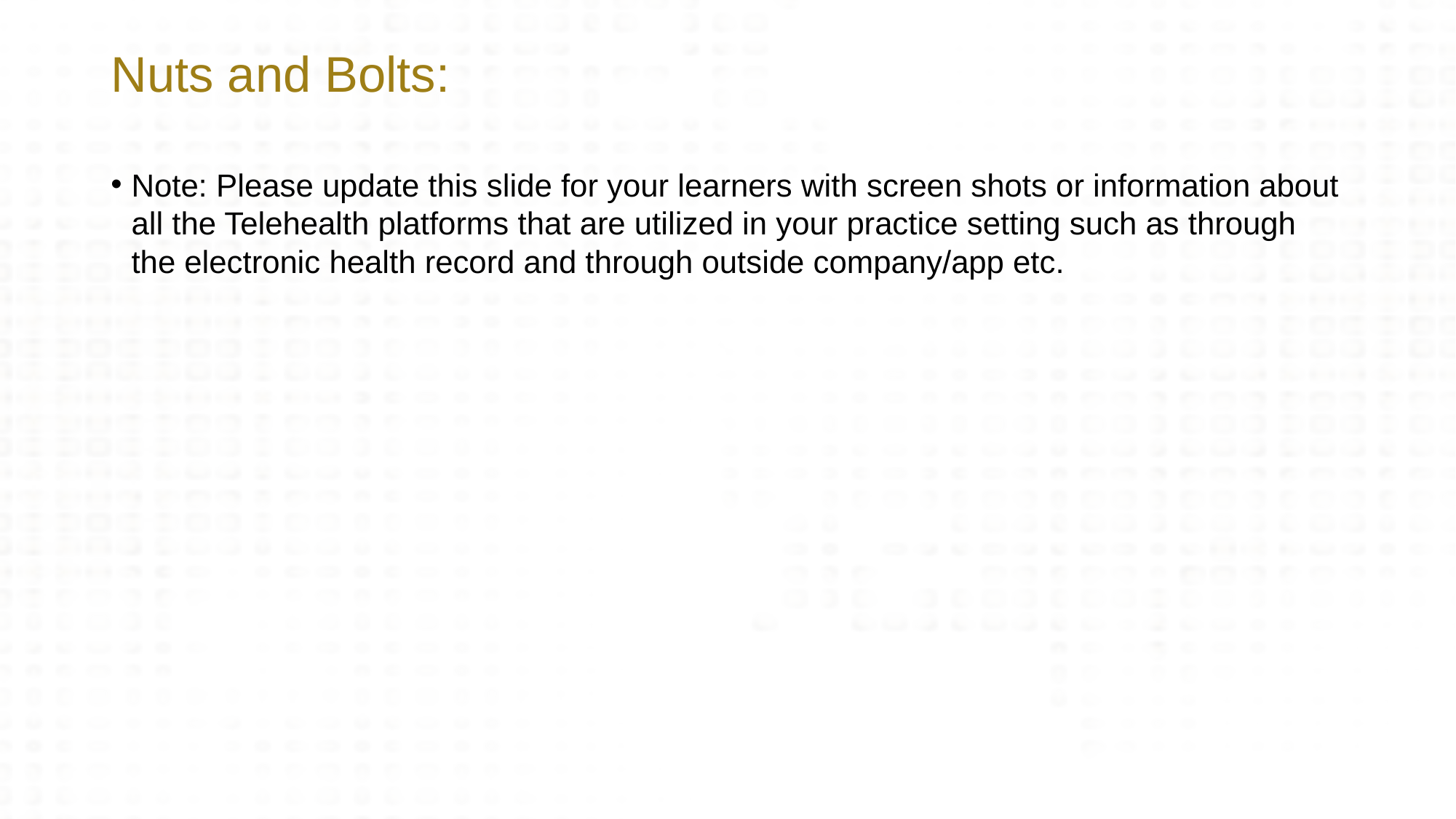

# Nuts and Bolts:
Note: Please update this slide for your learners with screen shots or information about all the Telehealth platforms that are utilized in your practice setting such as through the electronic health record and through outside company/app etc.

## Slide 4
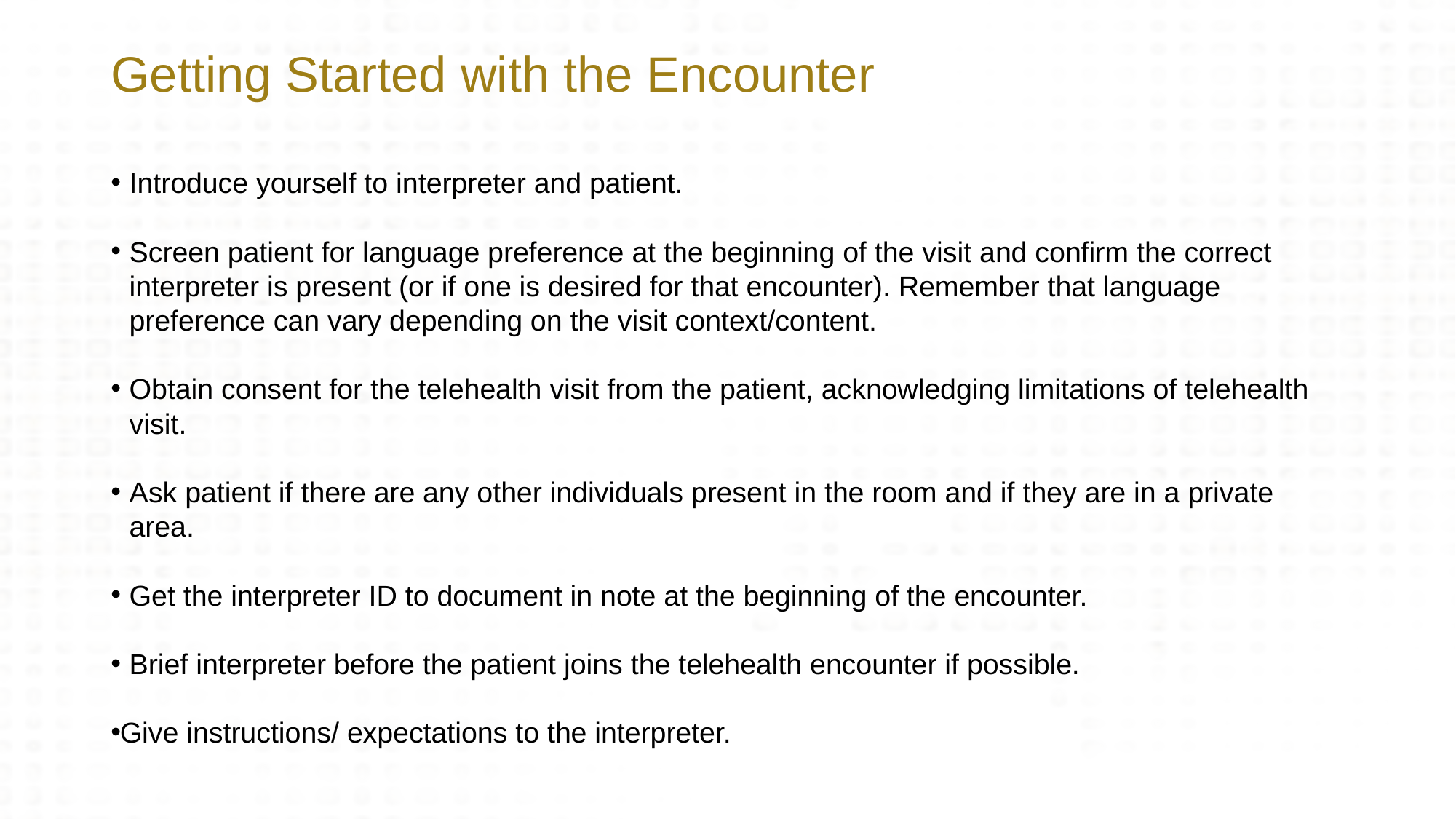

# Getting Started with the Encounter
Introduce yourself to interpreter and patient.
Screen patient for language preference at the beginning of the visit and confirm the correct interpreter is present (or if one is desired for that encounter). Remember that language preference can vary depending on the visit context/content.
Obtain consent for the telehealth visit from the patient, acknowledging limitations of telehealth visit.
Ask patient if there are any other individuals present in the room and if they are in a private area.
Get the interpreter ID to document in note at the beginning of the encounter.
Brief interpreter before the patient joins the telehealth encounter if possible.
Give instructions/ expectations to the interpreter.

## Slide 5
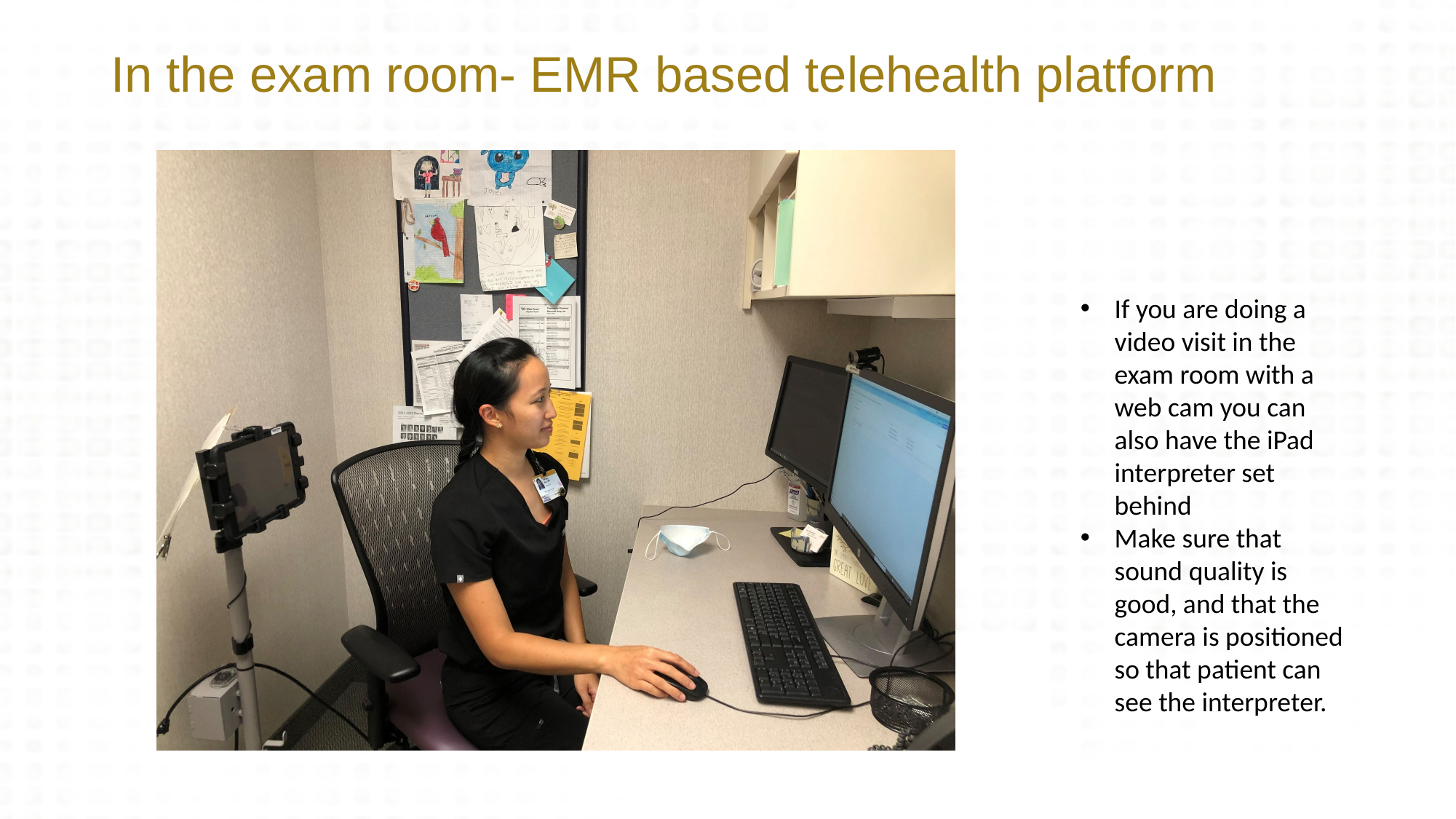

# In the exam room- EMR based telehealth platform
If you are doing a video visit in the exam room with a web cam you can also have the iPad interpreter set behind
Make sure that sound quality is good, and that the camera is positioned so that patient can see the interpreter.

## Slide 6
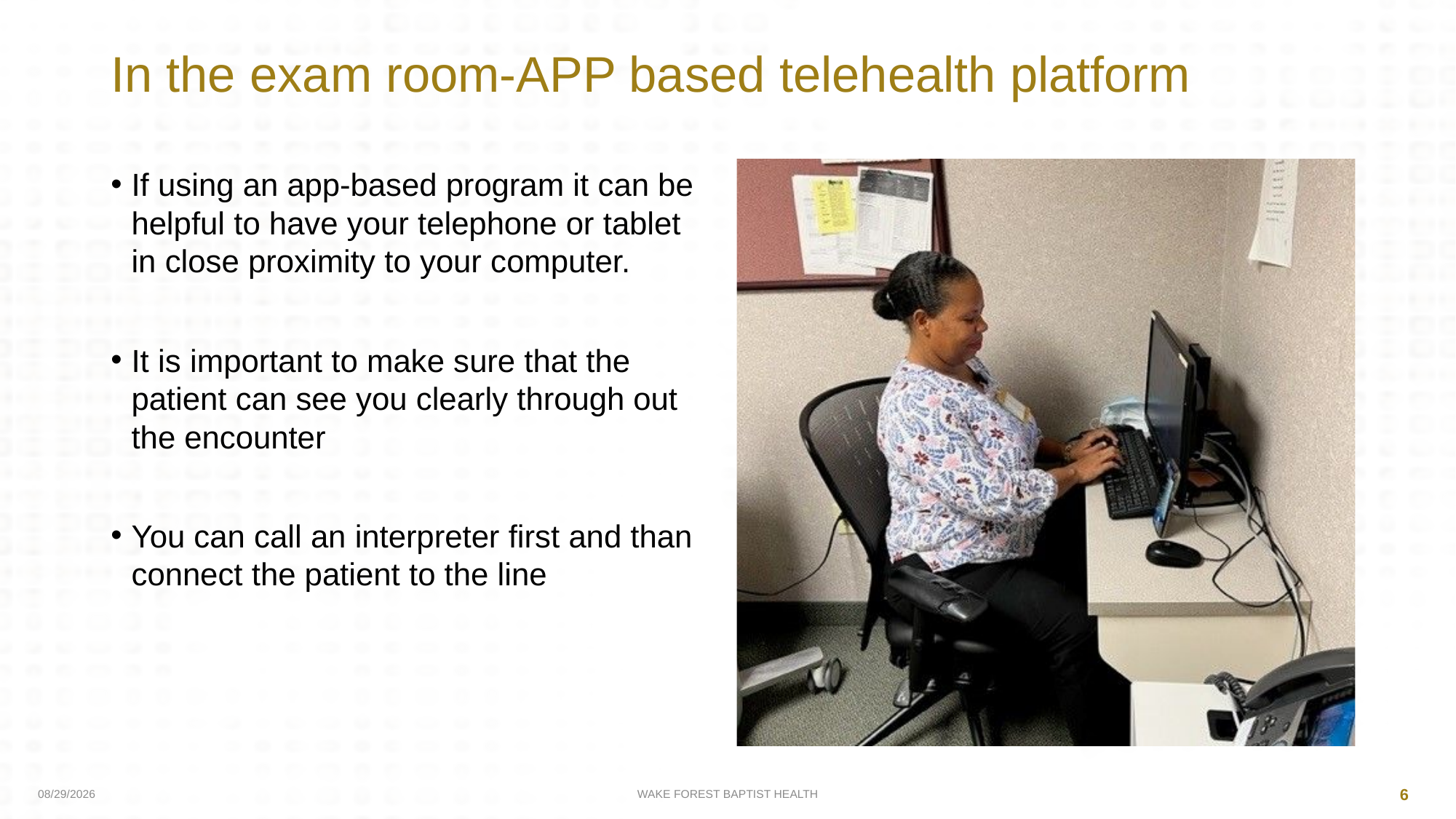

# In the exam room-APP based telehealth platform
If using an app-based program it can be helpful to have your telephone or tablet in close proximity to your computer.
It is important to make sure that the patient can see you clearly through out the encounter
You can call an interpreter first and than connect the patient to the line
6/16/2023
WAKE FOREST BAPTIST HEALTH
6

## Slide 7
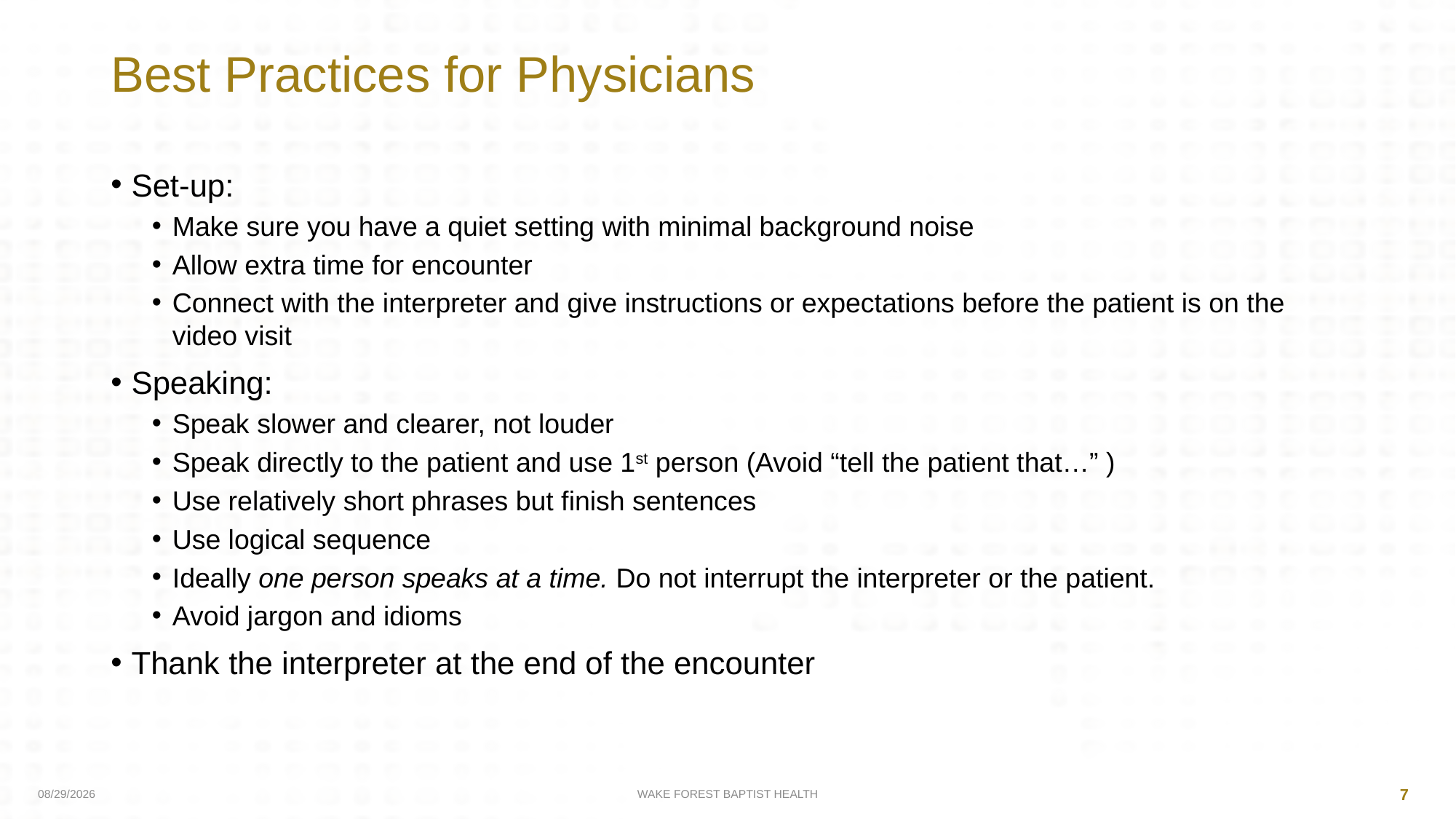

# Best Practices for Physicians
Set-up:
Make sure you have a quiet setting with minimal background noise
Allow extra time for encounter
Connect with the interpreter and give instructions or expectations before the patient is on the video visit
Speaking:
Speak slower and clearer, not louder
Speak directly to the patient and use 1st person (Avoid “tell the patient that…” )
Use relatively short phrases but finish sentences
Use logical sequence
Ideally one person speaks at a time. Do not interrupt the interpreter or the patient.
Avoid jargon and idioms
Thank the interpreter at the end of the encounter
6/16/2023
WAKE FOREST BAPTIST HEALTH
7

## Slide 8
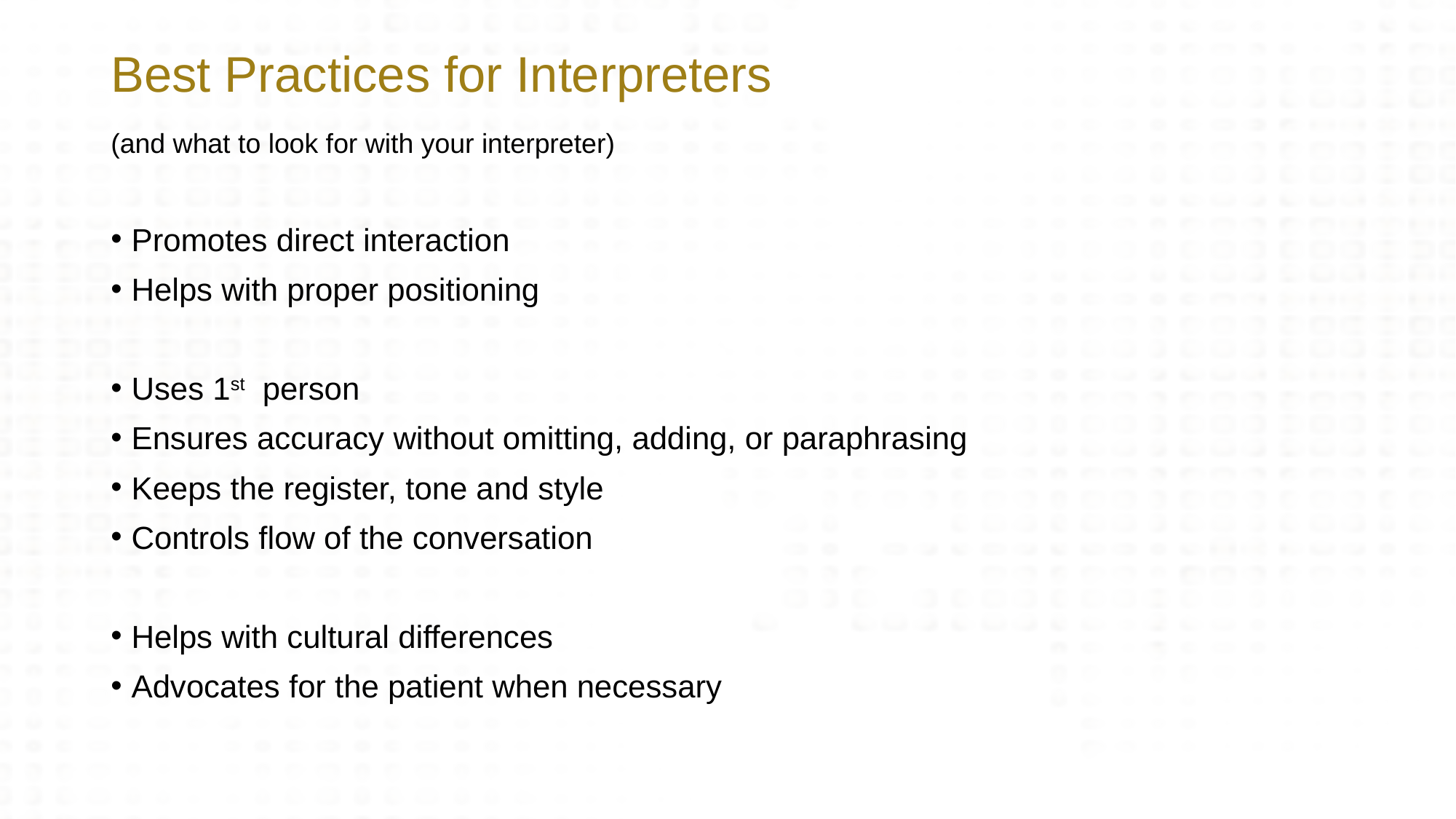

# Best Practices for Interpreters
(and what to look for with your interpreter)
Promotes direct interaction
Helps with proper positioning
Uses 1st person
Ensures accuracy without omitting, adding, or paraphrasing
Keeps the register, tone and style
Controls flow of the conversation
Helps with cultural differences
Advocates for the patient when necessary

## Slide 9
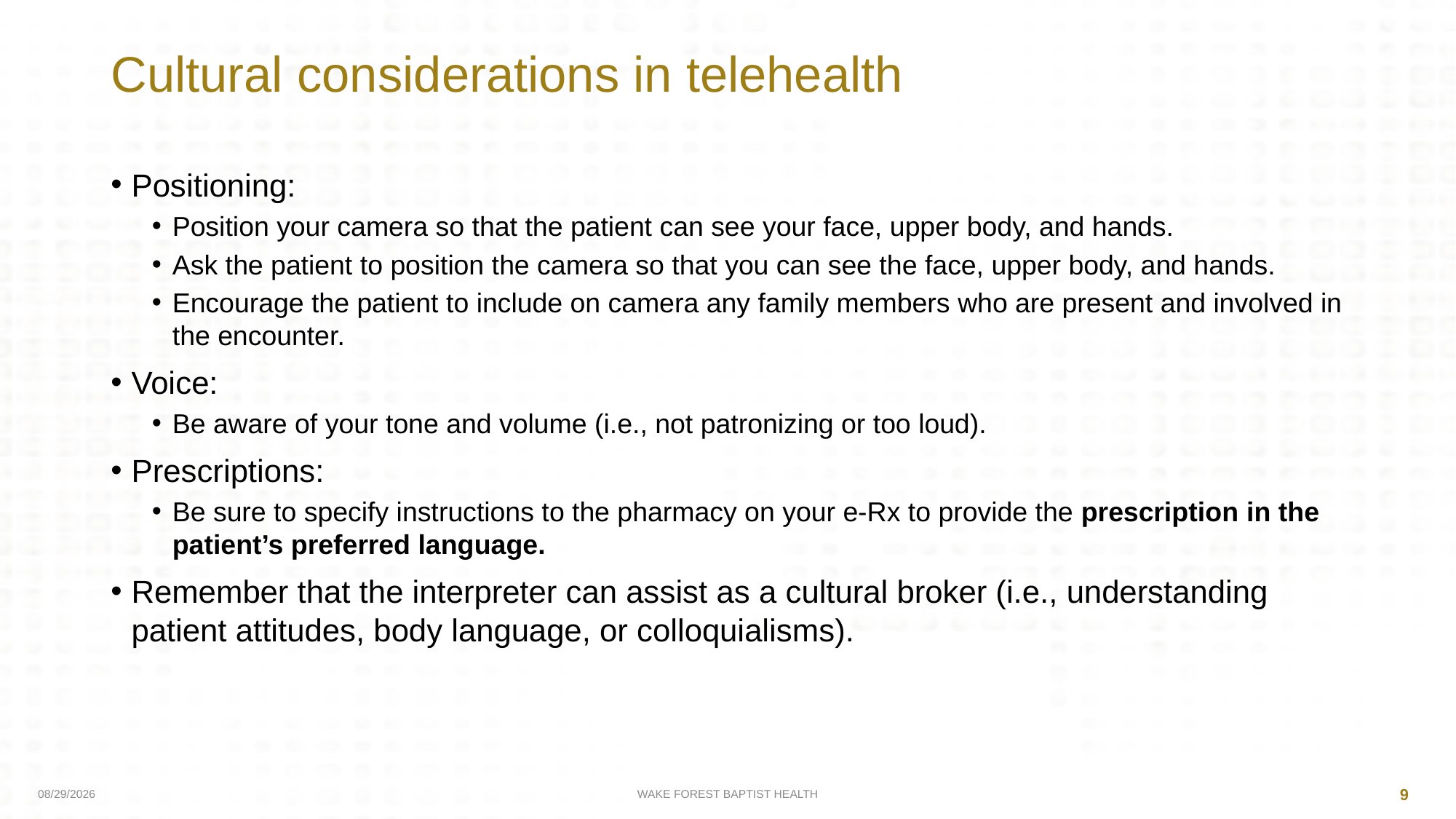

# Cultural considerations in telehealth
Positioning:
Position your camera so that the patient can see your face, upper body, and hands.
Ask the patient to position the camera so that you can see the face, upper body, and hands.
Encourage the patient to include on camera any family members who are present and involved in the encounter.
Voice:
Be aware of your tone and volume (i.e., not patronizing or too loud).
Prescriptions:
Be sure to specify instructions to the pharmacy on your e-Rx to provide the prescription in the patient’s preferred language.
Remember that the interpreter can assist as a cultural broker (i.e., understanding patient attitudes, body language, or colloquialisms).
6/16/2023
WAKE FOREST BAPTIST HEALTH
9

## Slide 10
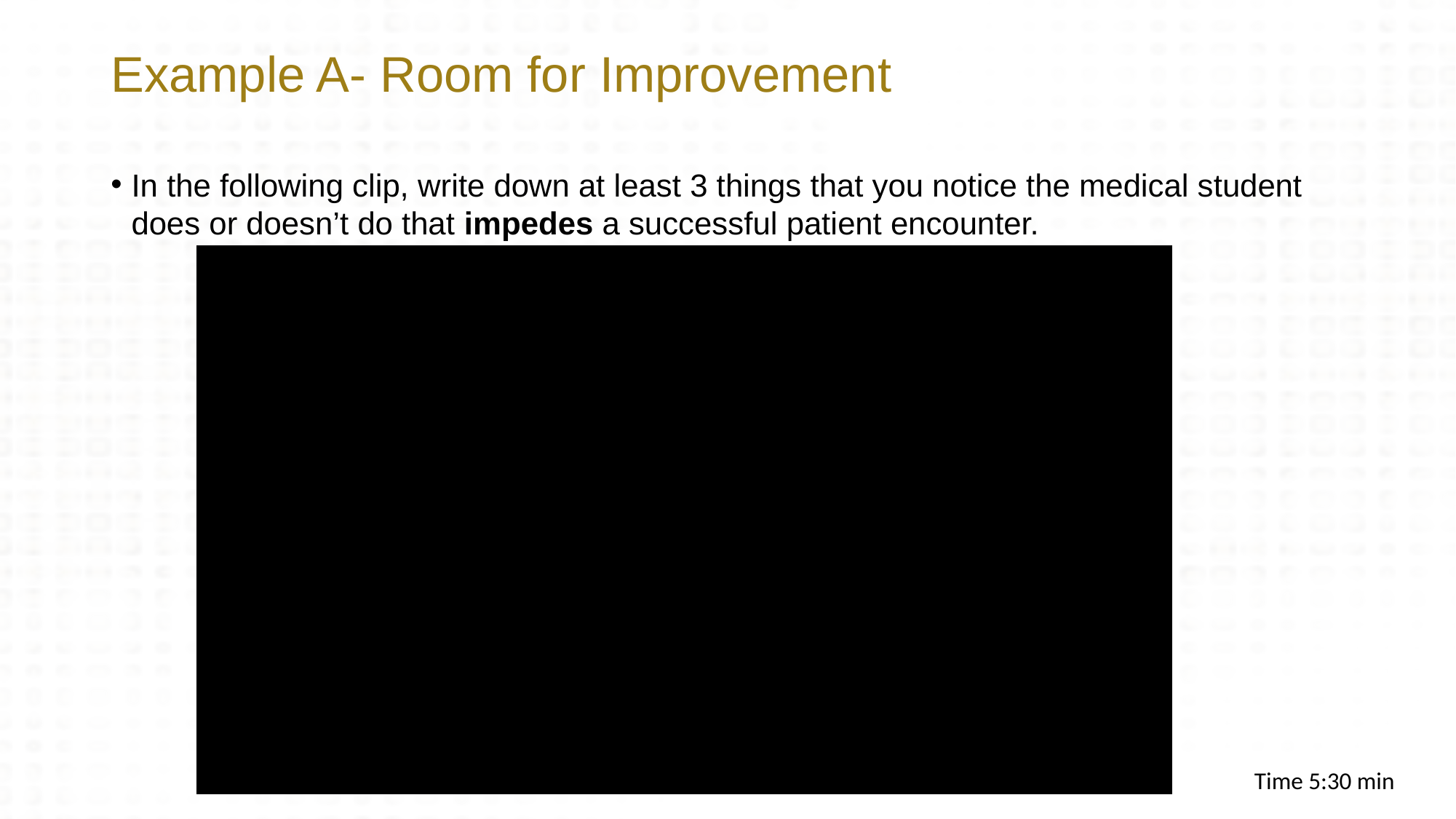

# Example A- Room for Improvement
In the following clip, write down at least 3 things that you notice the medical student does or doesn’t do that impedes a successful patient encounter.
Time 5:30 min

## Slide 11
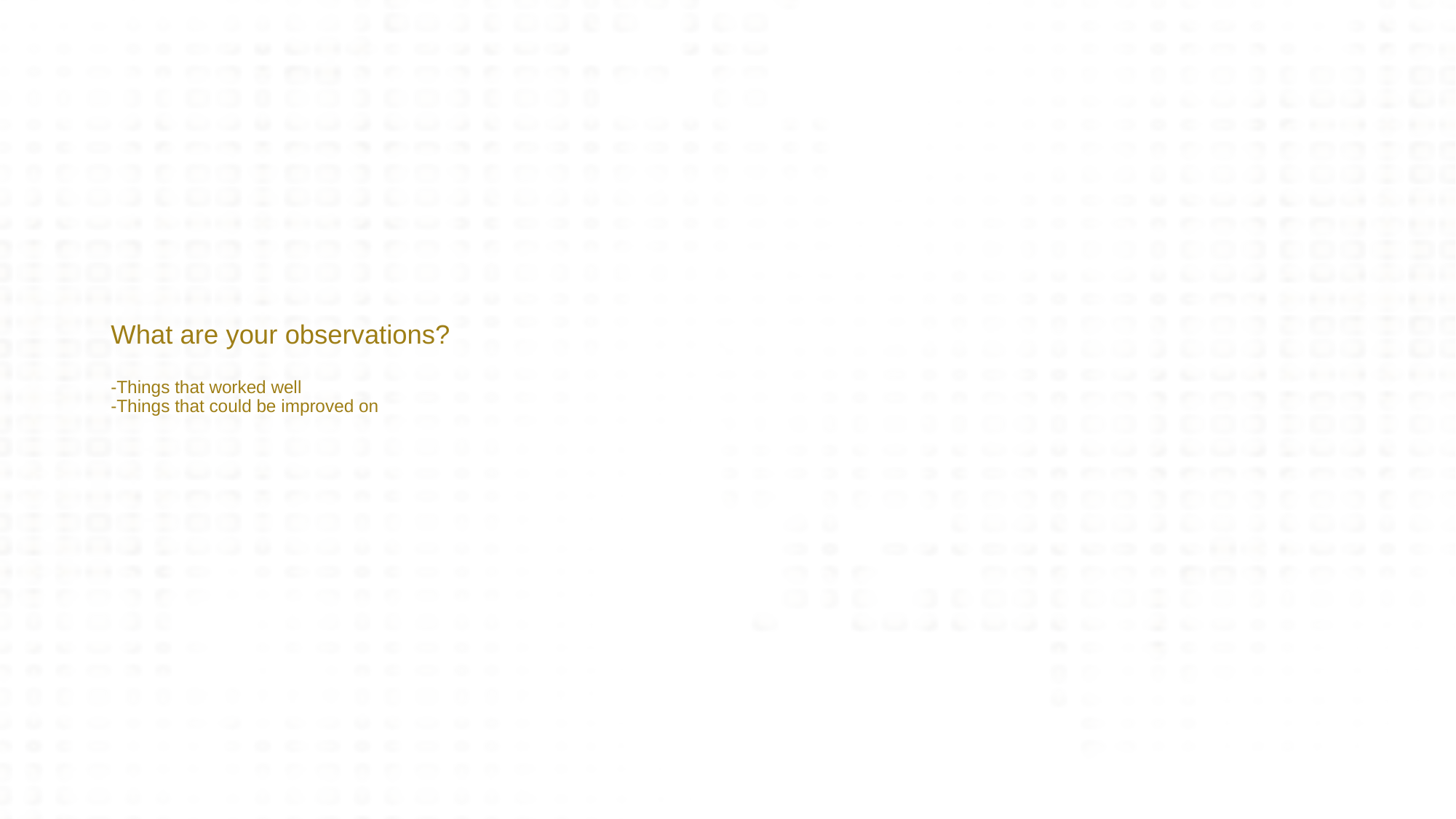

# What are your observations? -Things that worked well -Things that could be improved on

## Slide 12
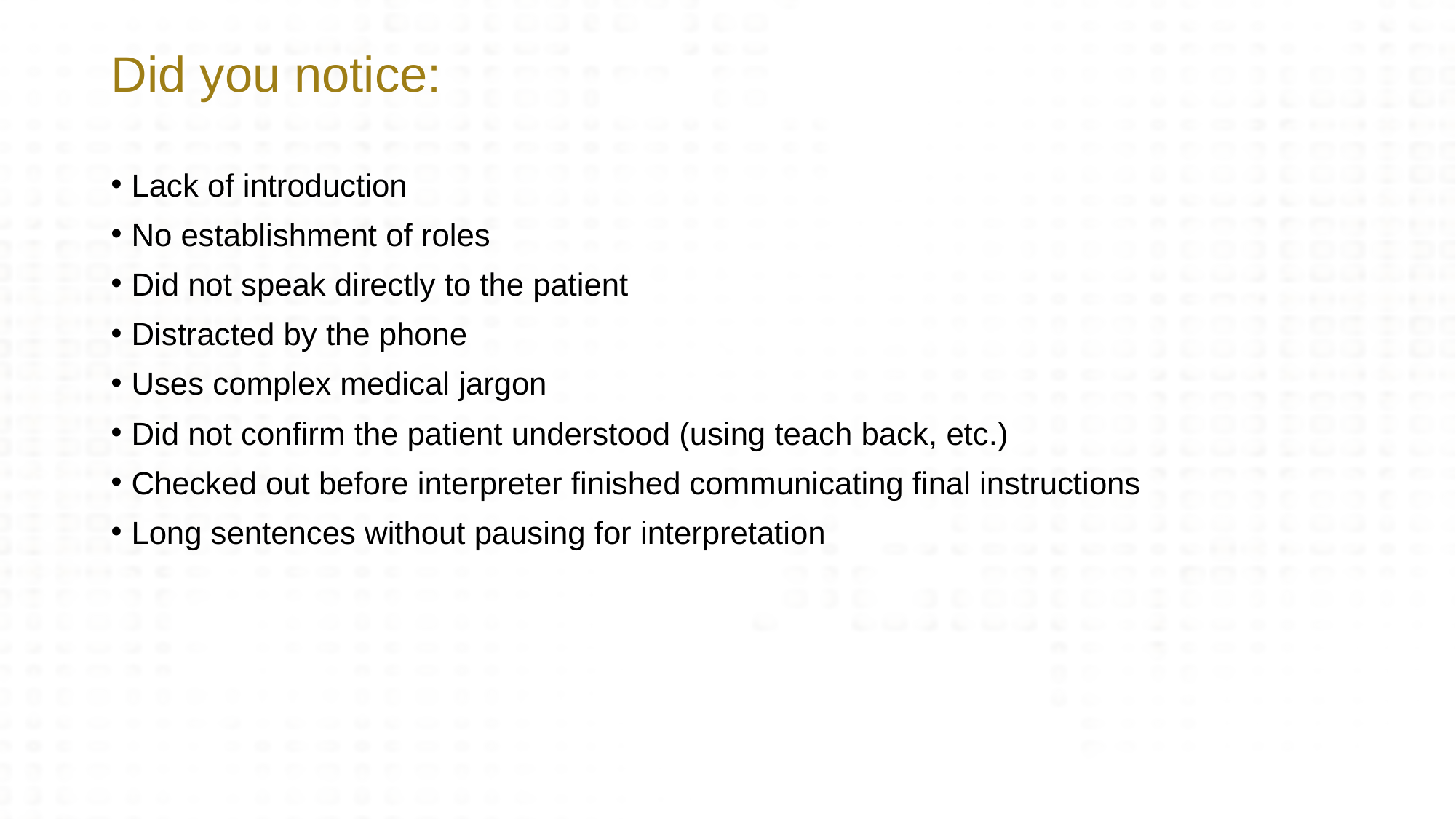

# Did you notice:
Lack of introduction
No establishment of roles
Did not speak directly to the patient
Distracted by the phone
Uses complex medical jargon
Did not confirm the patient understood (using teach back, etc.)
Checked out before interpreter finished communicating final instructions
Long sentences without pausing for interpretation

## Slide 13
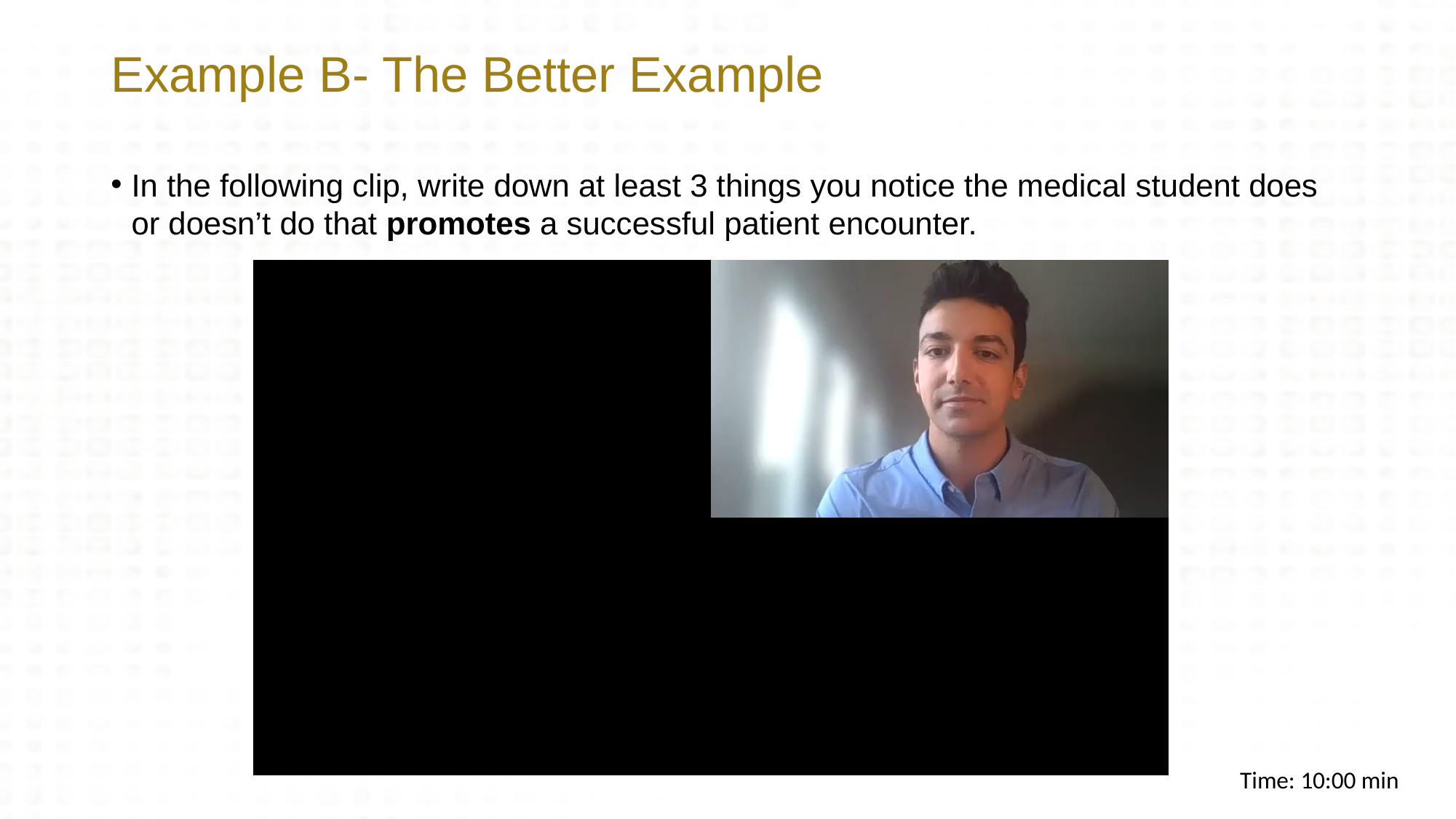

# Example B- The Better Example
In the following clip, write down at least 3 things you notice the medical student does or doesn’t do that promotes a successful patient encounter.
Time: 10:00 min

## Slide 14
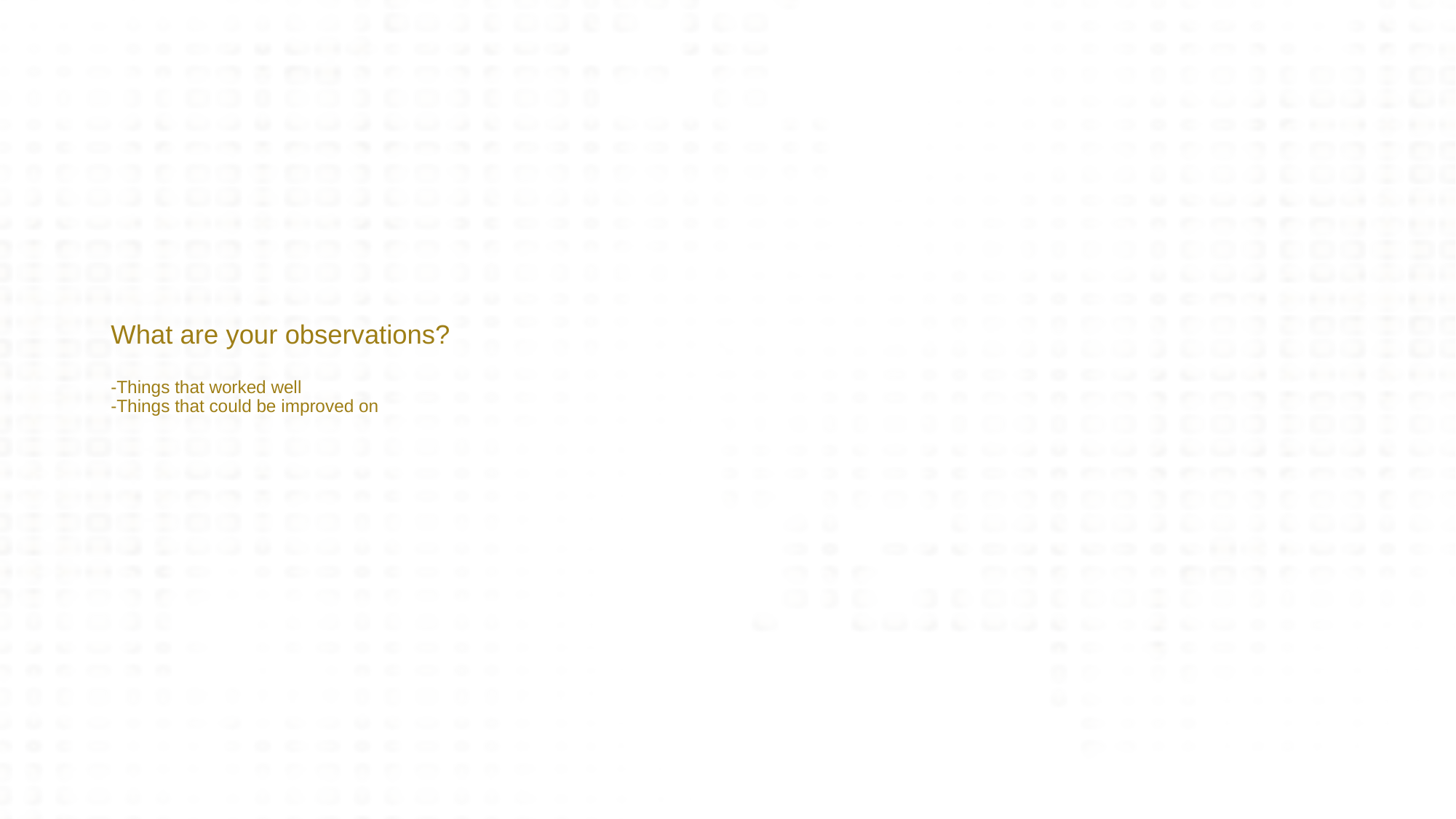

# What are your observations? -Things that worked well -Things that could be improved on

## Slide 15
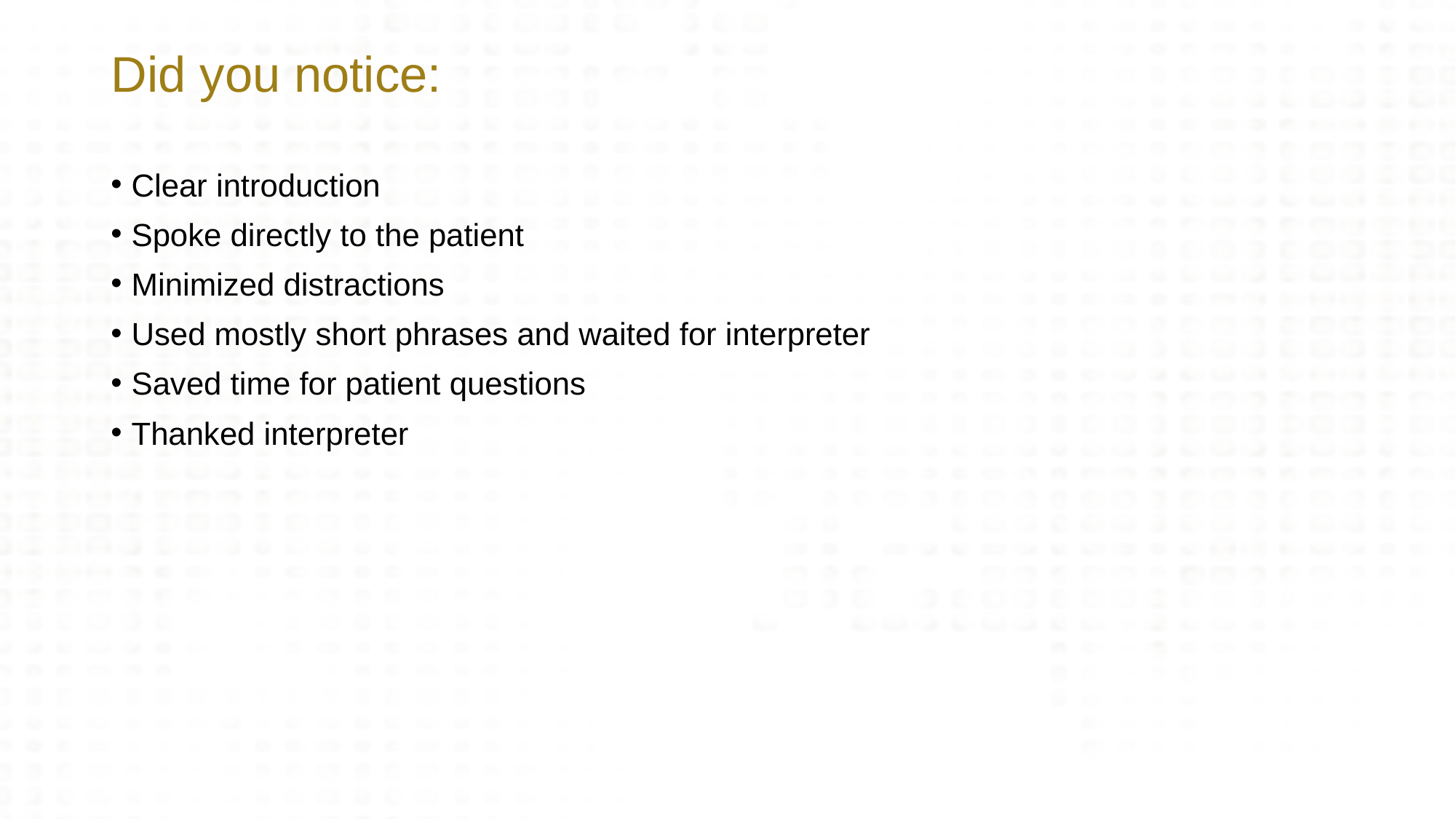

# Did you notice:
Clear introduction
Spoke directly to the patient
Minimized distractions
Used mostly short phrases and waited for interpreter
Saved time for patient questions
Thanked interpreter

## Slide 16
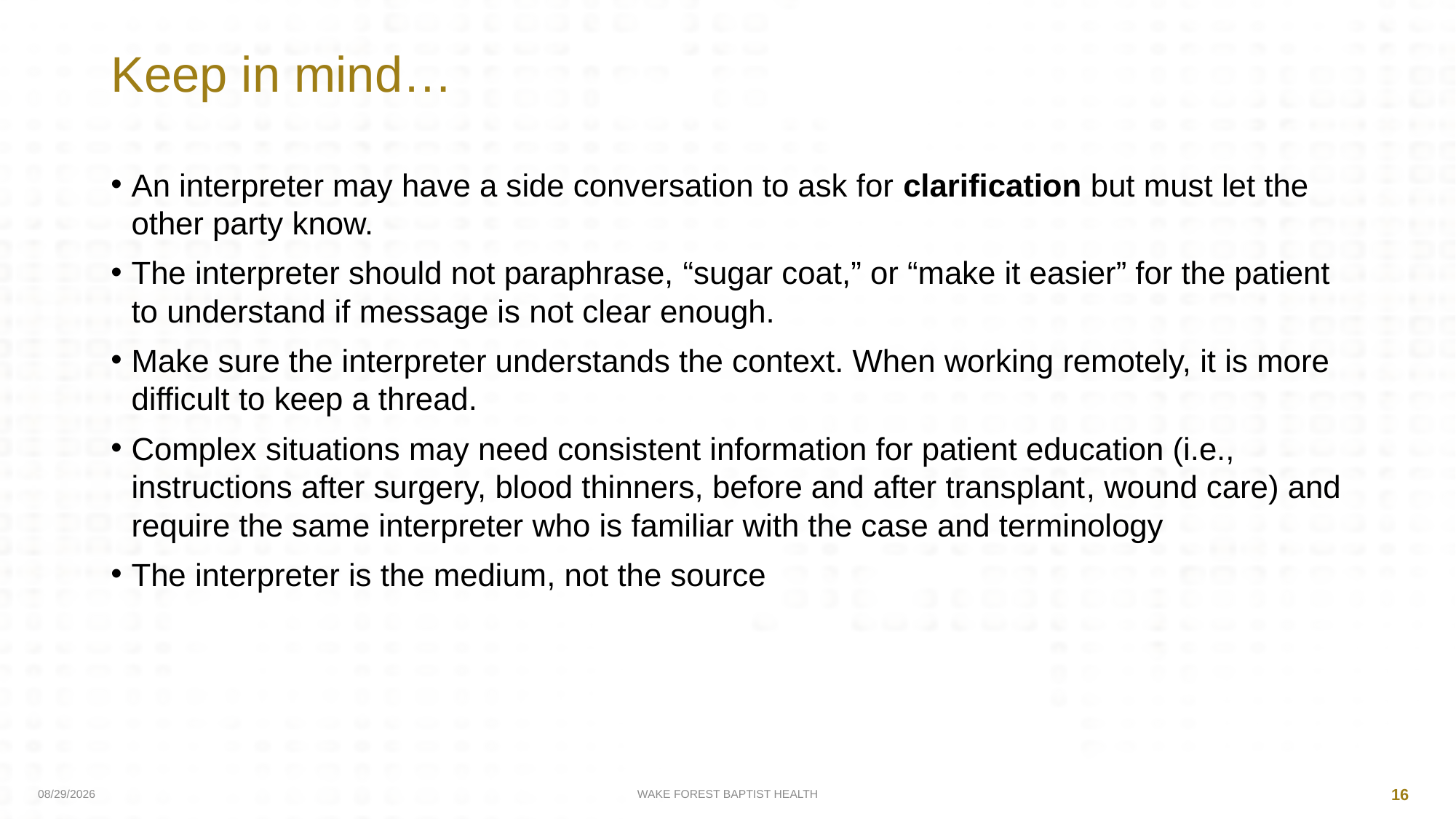

# Keep in mind…
An interpreter may have a side conversation to ask for clarification but must let the other party know.
The interpreter should not paraphrase, “sugar coat,” or “make it easier” for the patient to understand if message is not clear enough.
Make sure the interpreter understands the context. When working remotely, it is more difficult to keep a thread.
Complex situations may need consistent information for patient education (i.e., instructions after surgery, blood thinners, before and after transplant, wound care) and require the same interpreter who is familiar with the case and terminology
The interpreter is the medium, not the source
6/16/2023
WAKE FOREST BAPTIST HEALTH
16

## Slide 17
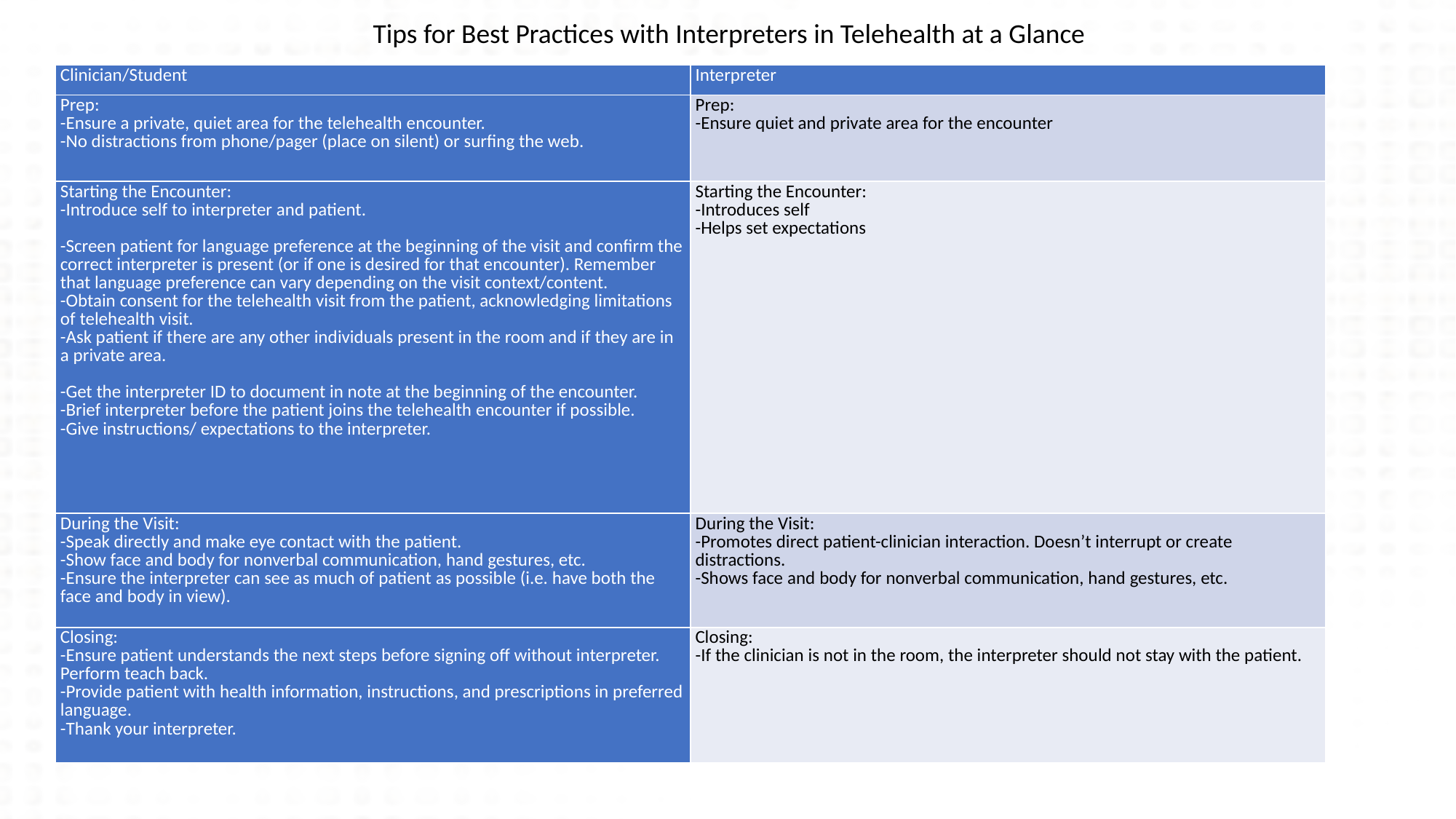

Tips for Best Practices with Interpreters in Telehealth at a Glance
| Clinician/Student | Interpreter |
| --- | --- |
| Prep: -Ensure a private, quiet area for the telehealth encounter. -No distractions from phone/pager (place on silent) or surfing the web. | Prep: -Ensure quiet and private area for the encounter |
| Starting the Encounter: -Introduce self to interpreter and patient. -Screen patient for language preference at the beginning of the visit and confirm the correct interpreter is present (or if one is desired for that encounter). Remember that language preference can vary depending on the visit context/content. -Obtain consent for the telehealth visit from the patient, acknowledging limitations of telehealth visit. -Ask patient if there are any other individuals present in the room and if they are in a private area. -Get the interpreter ID to document in note at the beginning of the encounter. -Brief interpreter before the patient joins the telehealth encounter if possible. -Give instructions/ expectations to the interpreter. | Starting the Encounter: -Introduces self -Helps set expectations |
| During the Visit: -Speak directly and make eye contact with the patient. -Show face and body for nonverbal communication, hand gestures, etc. -Ensure the interpreter can see as much of patient as possible (i.e. have both the face and body in view). | During the Visit: -Promotes direct patient-clinician interaction. Doesn’t interrupt or create distractions. -Shows face and body for nonverbal communication, hand gestures, etc. |
| Closing: -Ensure patient understands the next steps before signing off without interpreter. Perform teach back. -Provide patient with health information, instructions, and prescriptions in preferred language. -Thank your interpreter. | Closing: -If the clinician is not in the room, the interpreter should not stay with the patient. |

## Slide 18
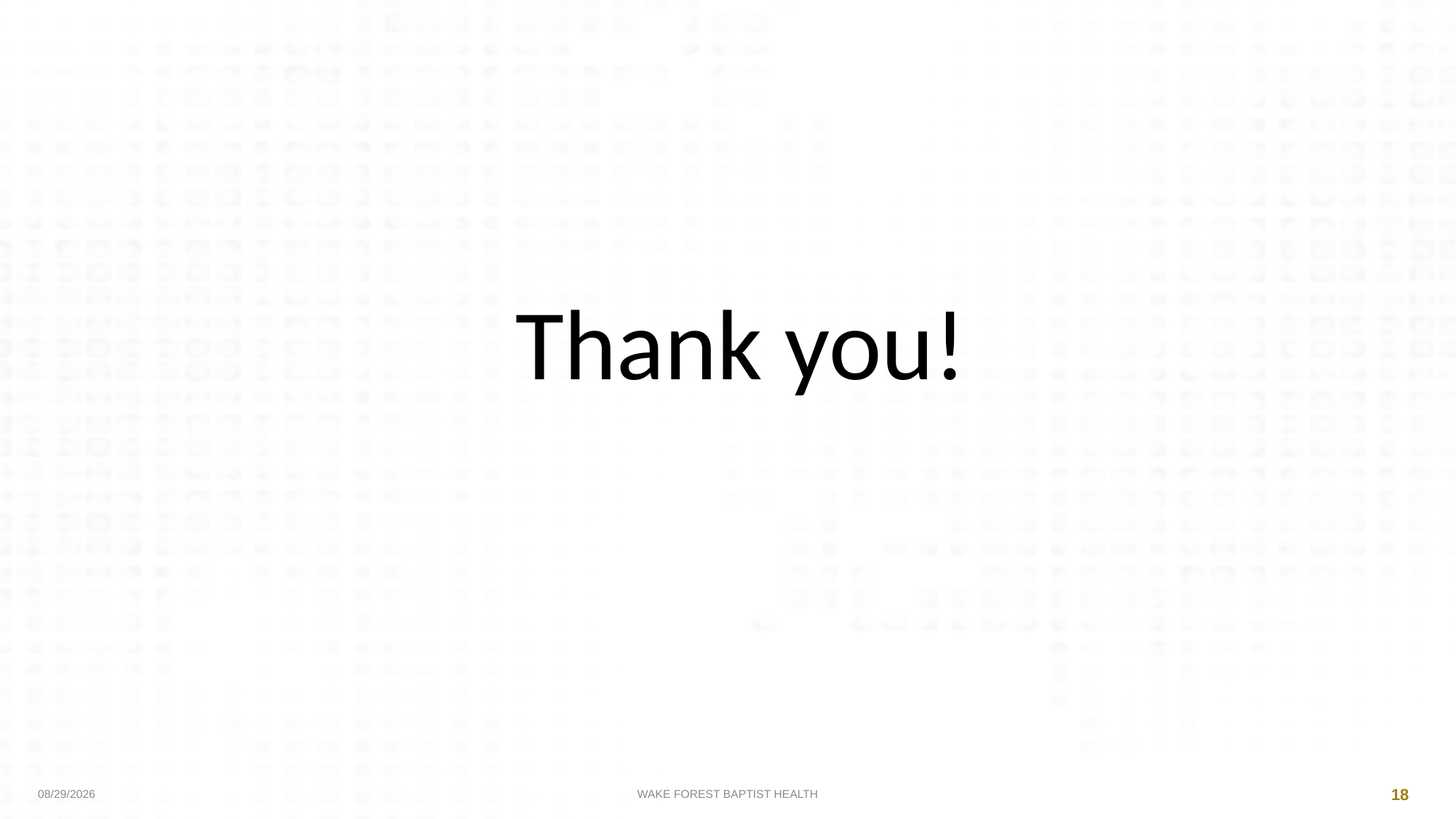

Thank you!
6/16/2023
WAKE FOREST BAPTIST HEALTH
18
