# Supplementary material for: Providing Equitable Care for Patients With Non-English Language Preference in Telemedicine: Training on Working With Interpreters in Telehealth
Source: MedEdPORTAL. 2023 Dec 14;19:11367. doi: 10.15766/mep_2374-8265.11367 (PMC10719426; doi:10.15766/mep_2374-8265.11367)
Supplement: Supplementary file 1 — Module Instructions.docxEquitable Care in Telemedicine folderFacilitator Guide for Alternative Teaching Options.docxInterpreter Room for Improvement Example.mp4Interpreter Better Example.mp4Working With Interpreters in Telehealth.pptxTips for Best Practices With Interpreters Handout.docxPostsurvey.docx [file mep_2374-8265.11367-s001.zip › G. Tips for Best Practices With Interpreters Handout.docx]

Appendix G: Tips for Best Practices with Interpreters Hand out

This is a one page handout that can be give to learners at the end of the session or made into a pocket card or QR code resource.

| **Clinician/Student** | **Interpreter** |
| --- | --- |
| **Prep:**  **-Ensure a private, quiet area for the telehealth encounter.**  **-No distractions from phone/pager (place on** **silent) or surfing the web.** | Prep:  -Ensure quiet and private area for the encounter |
| **Starting the Encounter:**  **-Introduce self to interpreter and patient.**  **-Screen** **patient for language preference at the beginning of the visit and confirm the correct interpreter is present (or if one is desired for that encounter). Remember that language preference can vary depending on the visit context/content.**  **-Obtain consent for the telehealth visit from the patient, acknowledging limitations of telehealth visit.**  **-Ask** **patient if there are any other individuals present in the room and if they are in** **a private area.**  **-Get the interpreter ID to document in note at the beginning of the encounter.**  **-Brief interpreter before the patient joins the telehealth encounter if possible.**  **-Give instructions/ expectations to the interpreter.** | Starting the Encounter: -Introduces self  -Helps set expectations |
| **During the Visit:**  **-Speak directly and make eye contact with the patient.**  **-Show face and body for nonverbal communication, hand gestures, etc.**  **-Ensure the interpreter can see as much of** **patient as possible (i.e., have both the face and body in view).** | During the Visit:  -Promotes direct patient-clinician interaction. Doesn’t interrupt or create distractions.  -Shows face and body for nonverbal communication, hand gestures, etc. |
| **Closing:**  **-Ensure patient understands the next steps before signing off without interpreter. Perform teach back.**  **-Provide** **patient with health information, instructions, and prescriptions in** **preferred language.**  **-Thank your interpreter.** | Closing:  -If the clinician is not in the room, the interpreter should not stay with the patient. |
