# Supplementary material for: Providing Equitable Care for Patients With Non-English Language Preference in Telemedicine: Training on Working With Interpreters in Telehealth
Source: MedEdPORTAL. 2023 Dec 14;19:11367. doi: 10.15766/mep_2374-8265.11367 (PMC10719426; doi:10.15766/mep_2374-8265.11367)
Supplement: Supplementary file 1 — Module Instructions.docxEquitable Care in Telemedicine folderFacilitator Guide for Alternative Teaching Options.docxInterpreter Room for Improvement Example.mp4Interpreter Better Example.mp4Working With Interpreters in Telehealth.pptxTips for Best Practices With Interpreters Handout.docxPostsurvey.docx [file mep_2374-8265.11367-s001.zip › H. Postsurvey.docx]

Appendix H: Post-Survey Questions- Interpreters in Telehealth Module

1. Was the amount of time required to complete this lesson manageable?
   - Yes
   - No
2. How effective was this lesson in teaching the nuts and bolts of how to access interpreters in the telehealth settings?
   - Extremely effective
   - Quite effective
   - Moderately effective
   - Slightly effective
   - Not at all effective
3. How effective was this lesson in conveying how to effectively work with interpreters in telehealth encounters?
   - Extremely effective
   - Quite effective
   - Moderately effective
   - Slightly effective
   - Not at all effective
4. How effective was this lesson in increasing your comfort with patient visits with non-English speaking patients?
   - Extremely effective
   - Quite effective
   - Moderately effective
   - Slightly effective
   - Not at all effective
5. I would recommend this lesson to future students
   - Yes
   - Yes, with some changes
   - No
6. Please share any comments or feedback
