# Supplementary figures and images for: Providing Equitable Care for Patients With Non-English Language Preference in Telemedicine: Training on Working With Interpreters in Telehealth
Source: MedEdPORTAL. 2023 Dec 14;19:11367. doi: 10.15766/mep_2374-8265.11367 (PMC10719426; doi:10.15766/mep_2374-8265.11367)

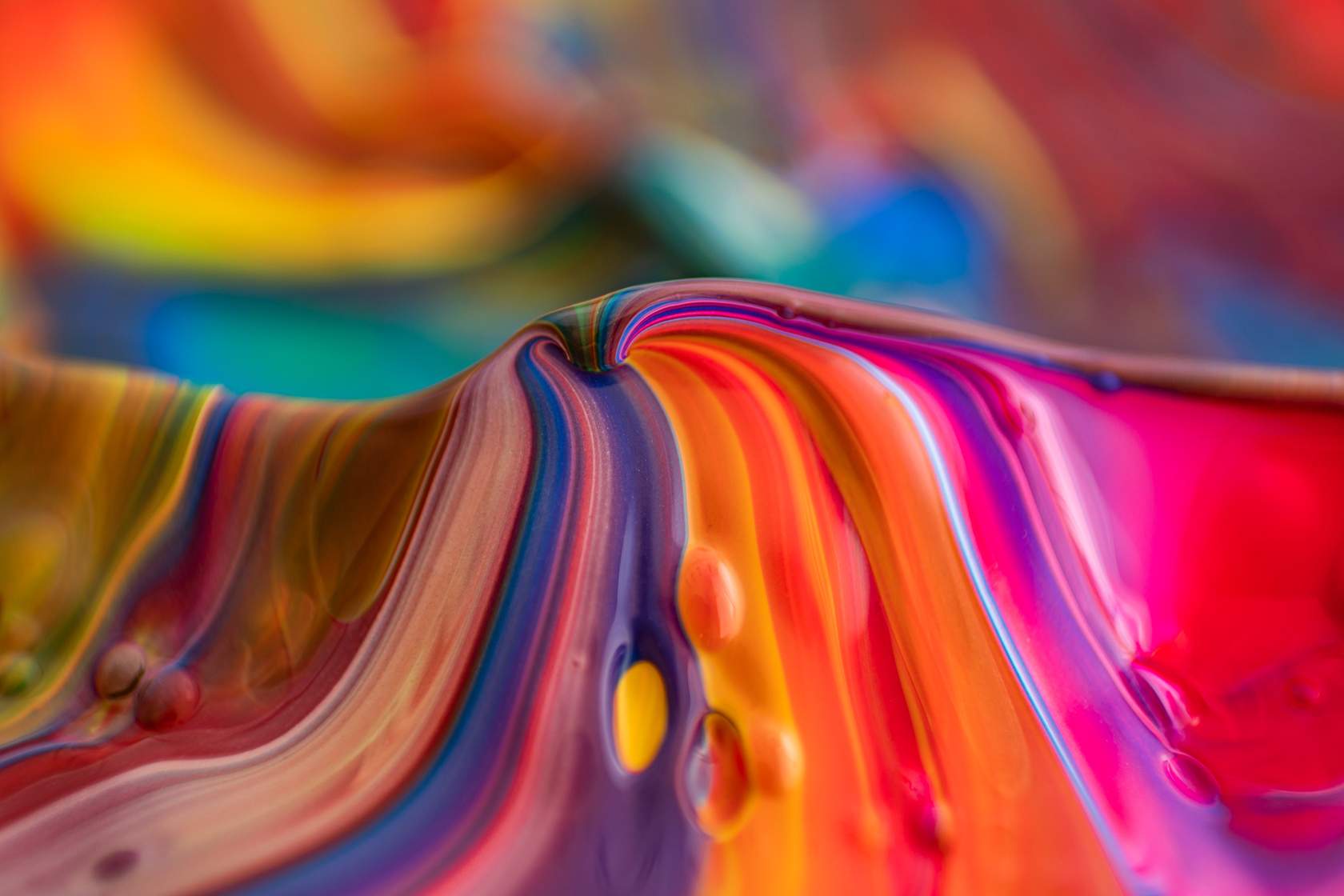

Supplement: Supplementary file 1 — Module Instructions.docxEquitable Care in Telemedicine folderFacilitator Guide for Alternative Teaching Options.docxInterpreter Room for Improvement Example.mp4Interpreter Better Example.mp4Working With Interpreters in Telehealth.pptxTips for Best Practices With Interpreters Handout.docxPostsurvey.docx [file mep_2374-8265.11367-s001.zip › B. Equitable Care in Telemedicine/content/assets/3c0YOV4vnSlGOiFV_eNQh042oDyaW--D2.jpg]

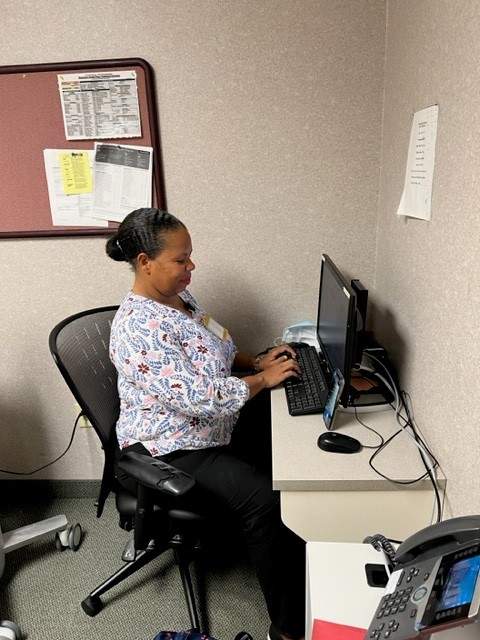

Supplement: Supplementary file 1 — Module Instructions.docxEquitable Care in Telemedicine folderFacilitator Guide for Alternative Teaching Options.docxInterpreter Room for Improvement Example.mp4Interpreter Better Example.mp4Working With Interpreters in Telehealth.pptxTips for Best Practices With Interpreters Handout.docxPostsurvey.docx [file mep_2374-8265.11367-s001.zip › B. Equitable Care in Telemedicine/content/assets/3I1Jps4T6gl_229P_-Itmdsl3ADVwTamB.jpg]

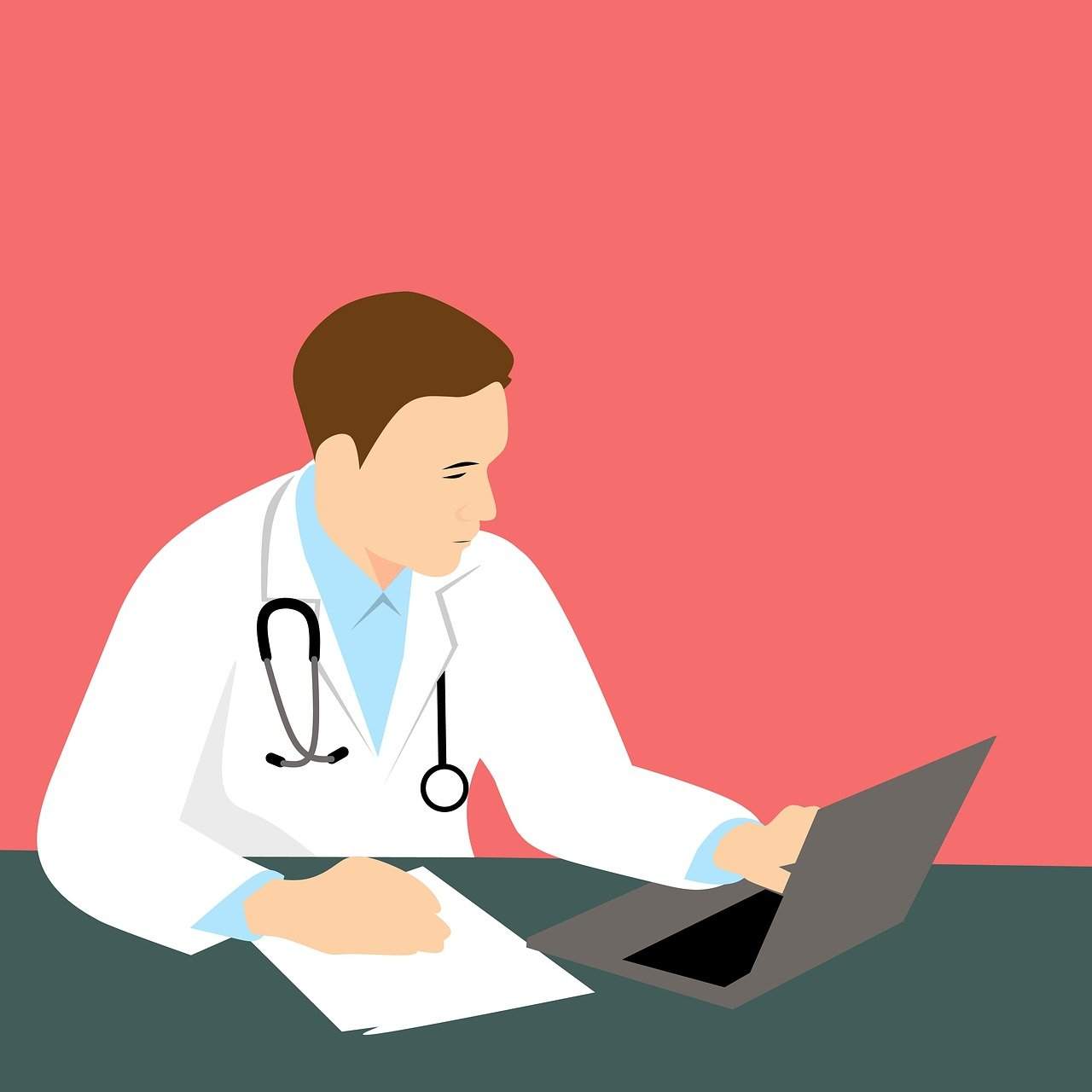

Supplement: Supplementary file 1 — Module Instructions.docxEquitable Care in Telemedicine folderFacilitator Guide for Alternative Teaching Options.docxInterpreter Room for Improvement Example.mp4Interpreter Better Example.mp4Working With Interpreters in Telehealth.pptxTips for Best Practices With Interpreters Handout.docxPostsurvey.docx [file mep_2374-8265.11367-s001.zip › B. Equitable Care in Telemedicine/content/assets/3qcHvjNv6NrAZUVK_BBSGMEUnCdHHuqEL.jpg]

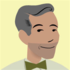

Supplement: Supplementary file 1 — Module Instructions.docxEquitable Care in Telemedicine folderFacilitator Guide for Alternative Teaching Options.docxInterpreter Room for Improvement Example.mp4Interpreter Better Example.mp4Working With Interpreters in Telehealth.pptxTips for Best Practices With Interpreters Handout.docxPostsurvey.docx [file mep_2374-8265.11367-s001.zip › B. Equitable Care in Telemedicine/content/assets/3Tx1er2Hzi8DI5H-_small_1412405266.png]

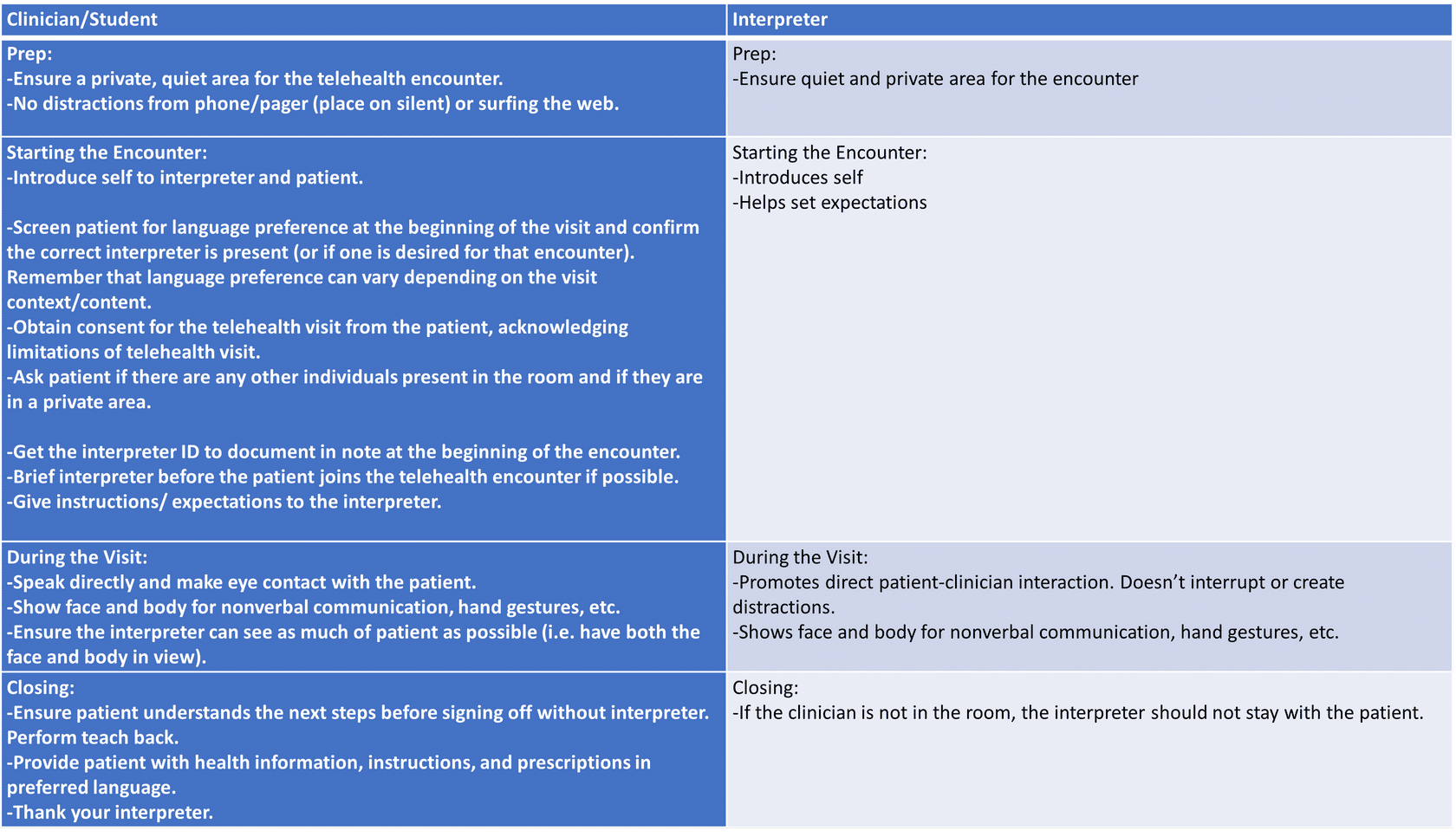

Supplement: Supplementary file 1 — Module Instructions.docxEquitable Care in Telemedicine folderFacilitator Guide for Alternative Teaching Options.docxInterpreter Room for Improvement Example.mp4Interpreter Better Example.mp4Working With Interpreters in Telehealth.pptxTips for Best Practices With Interpreters Handout.docxPostsurvey.docx [file mep_2374-8265.11367-s001.zip › B. Equitable Care in Telemedicine/content/assets/4eFZAwIxmdKNoEGj_e9-UZAxAA51GK6gW.png]

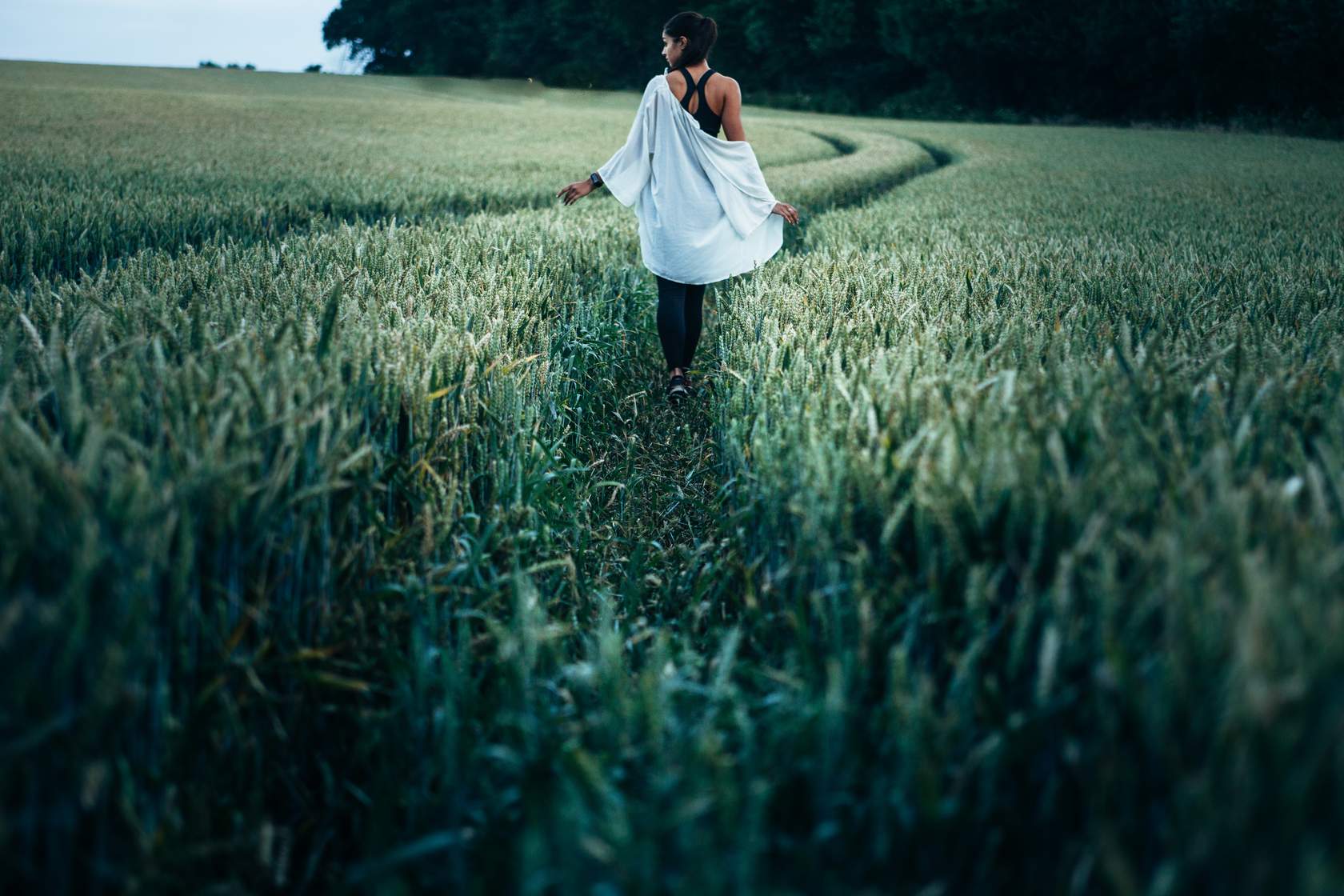

Supplement: Supplementary file 1 — Module Instructions.docxEquitable Care in Telemedicine folderFacilitator Guide for Alternative Teaching Options.docxInterpreter Room for Improvement Example.mp4Interpreter Better Example.mp4Working With Interpreters in Telehealth.pptxTips for Best Practices With Interpreters Handout.docxPostsurvey.docx [file mep_2374-8265.11367-s001.zip › B. Equitable Care in Telemedicine/content/assets/B1zBQLz8Ycg4GzMd_field.jpg]

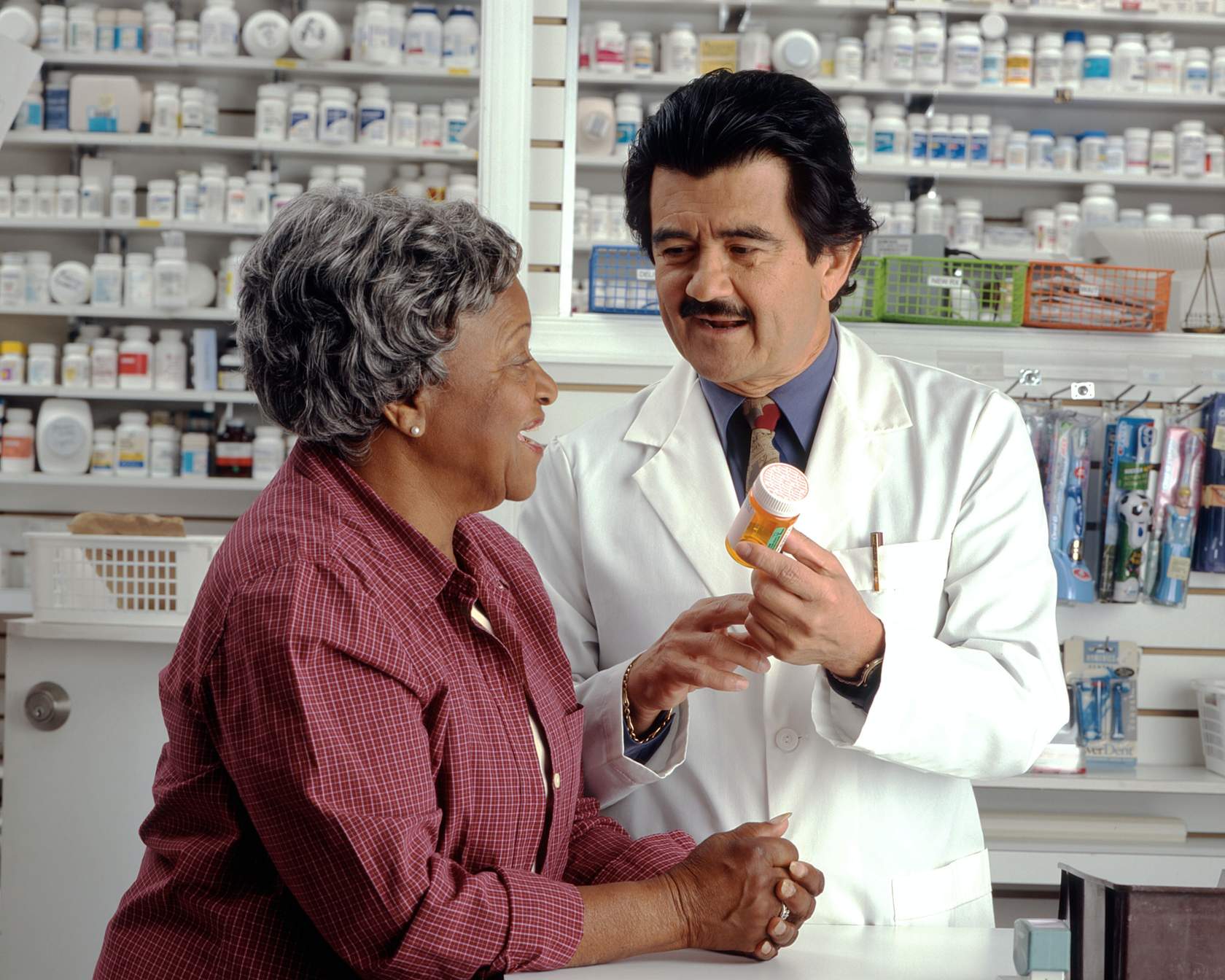

Supplement: Supplementary file 1 — Module Instructions.docxEquitable Care in Telemedicine folderFacilitator Guide for Alternative Teaching Options.docxInterpreter Room for Improvement Example.mp4Interpreter Better Example.mp4Working With Interpreters in Telehealth.pptxTips for Best Practices With Interpreters Handout.docxPostsurvey.docx [file mep_2374-8265.11367-s001.zip › B. Equitable Care in Telemedicine/content/assets/c0JzDB6UAKeUoPgj_8nBajZi3xNJcVJbS.jpg]

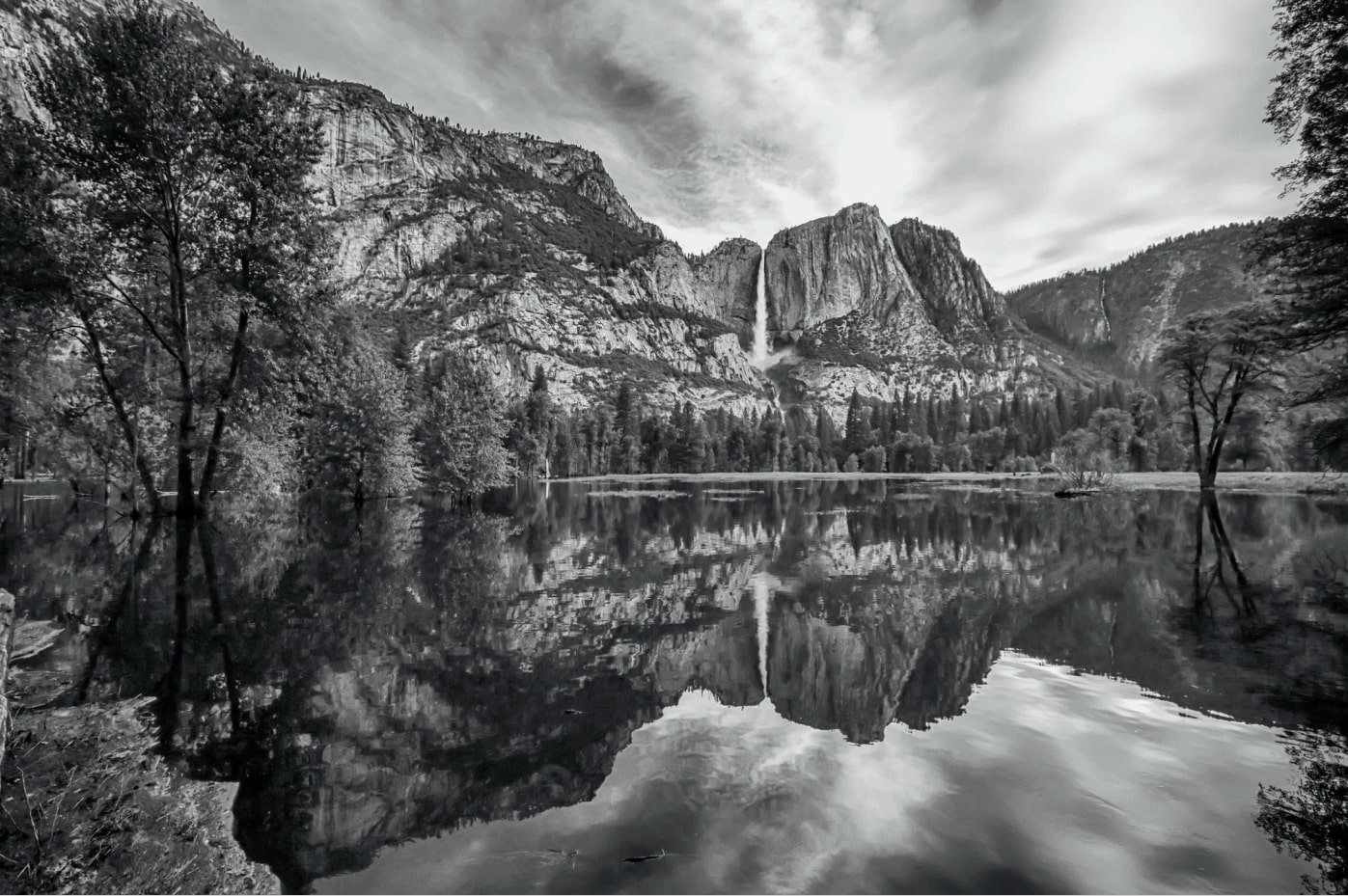

Supplement: Supplementary file 1 — Module Instructions.docxEquitable Care in Telemedicine folderFacilitator Guide for Alternative Teaching Options.docxInterpreter Room for Improvement Example.mp4Interpreter Better Example.mp4Working With Interpreters in Telehealth.pptxTips for Best Practices With Interpreters Handout.docxPostsurvey.docx [file mep_2374-8265.11367-s001.zip › B. Equitable Care in Telemedicine/content/assets/g_eg9lOfaut5Opam_example-header-image.jpg]

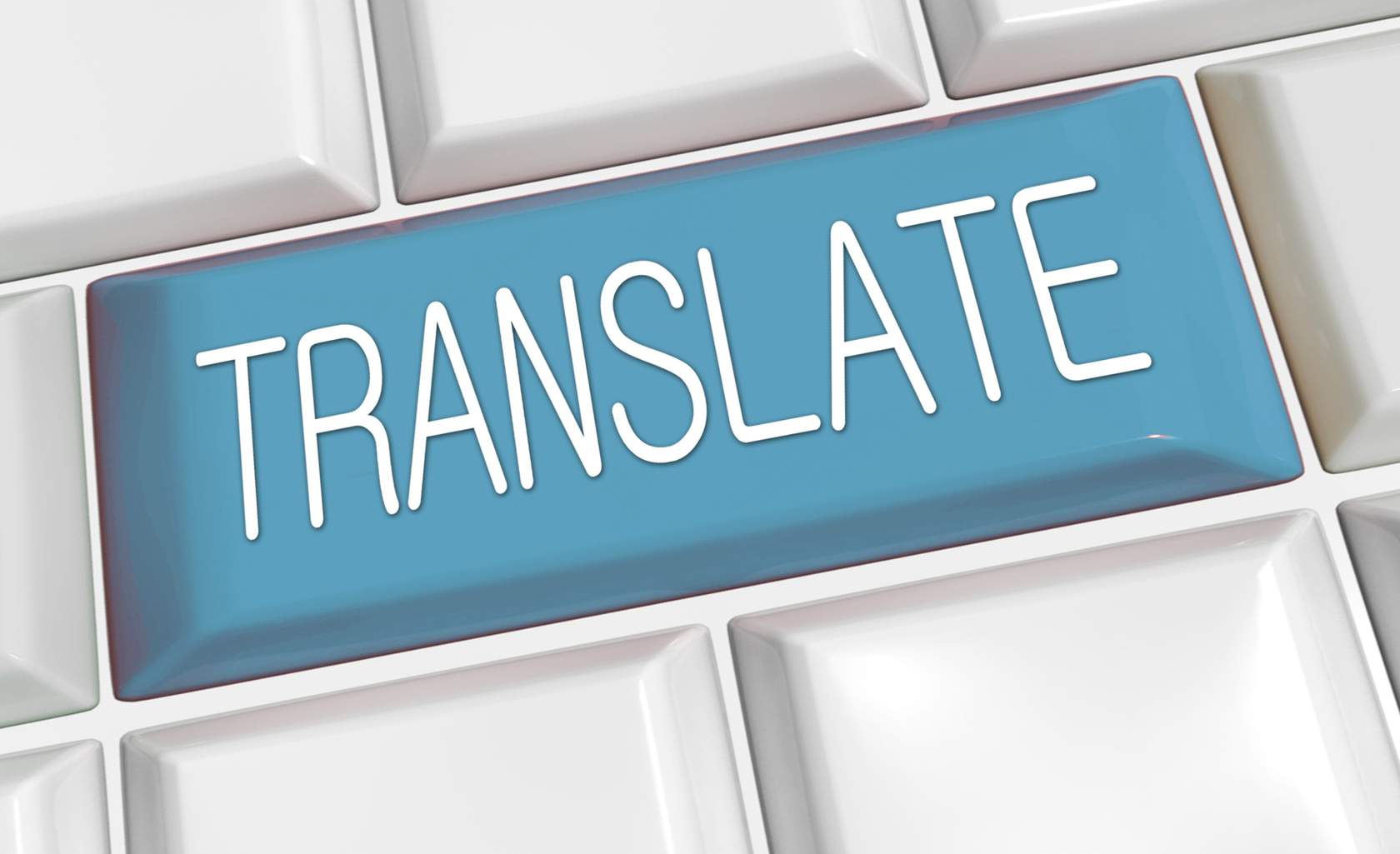

Supplement: Supplementary file 1 — Module Instructions.docxEquitable Care in Telemedicine folderFacilitator Guide for Alternative Teaching Options.docxInterpreter Room for Improvement Example.mp4Interpreter Better Example.mp4Working With Interpreters in Telehealth.pptxTips for Best Practices With Interpreters Handout.docxPostsurvey.docx [file mep_2374-8265.11367-s001.zip › B. Equitable Care in Telemedicine/content/assets/g_KPMweq4dSXDK1m_IQTMYWVfC7eDT0Bk.jpg]

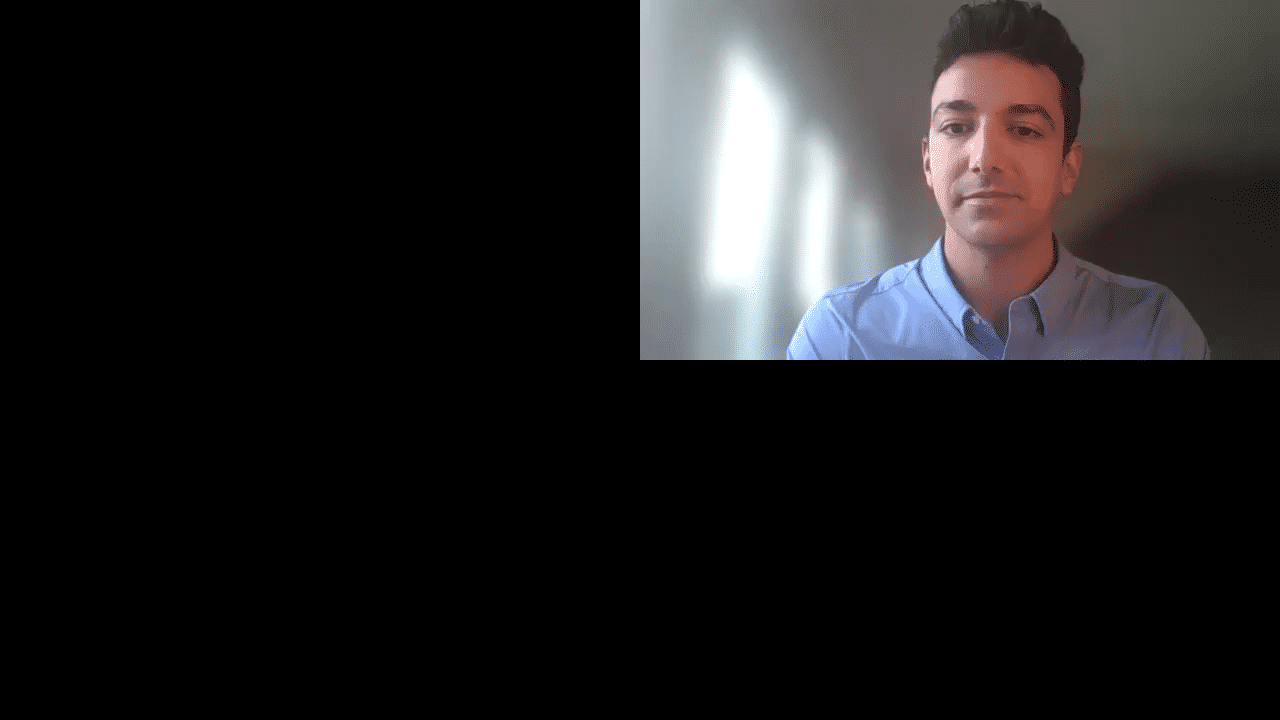

Supplement: Supplementary file 1 — Module Instructions.docxEquitable Care in Telemedicine folderFacilitator Guide for Alternative Teaching Options.docxInterpreter Room for Improvement Example.mp4Interpreter Better Example.mp4Working With Interpreters in Telehealth.pptxTips for Best Practices With Interpreters Handout.docxPostsurvey.docx [file mep_2374-8265.11367-s001.zip › B. Equitable Care in Telemedicine/content/assets/Gp_Ut1gvCcmaFZAN_transcoded-zyTWTFGNkU0rczjk-telehealth-good-example-00001.png]

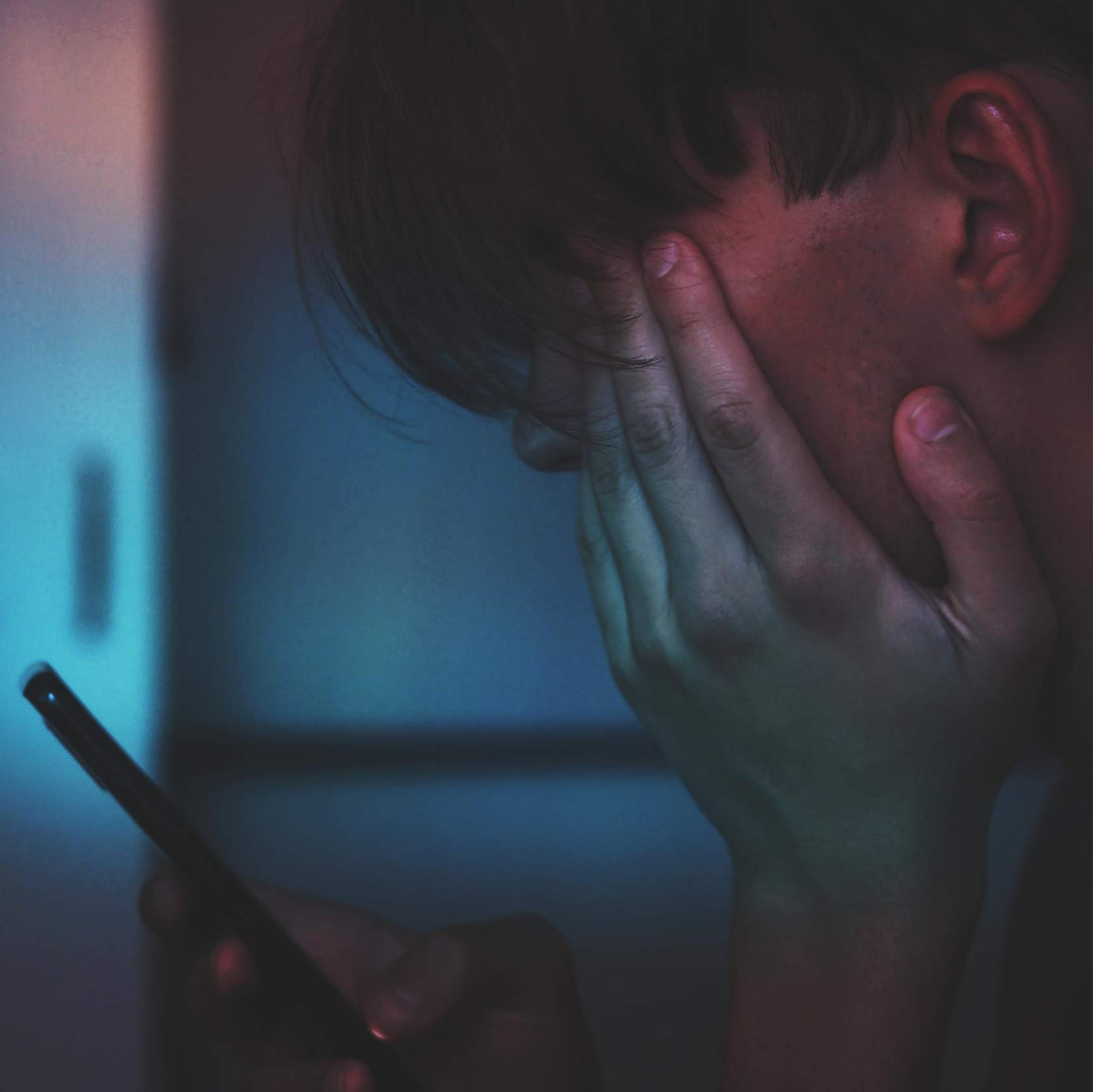

Supplement: Supplementary file 1 — Module Instructions.docxEquitable Care in Telemedicine folderFacilitator Guide for Alternative Teaching Options.docxInterpreter Room for Improvement Example.mp4Interpreter Better Example.mp4Working With Interpreters in Telehealth.pptxTips for Best Practices With Interpreters Handout.docxPostsurvey.docx [file mep_2374-8265.11367-s001.zip › B. Equitable Care in Telemedicine/content/assets/HLunfN5o8go-flD8_VNEQywRCFURmnFWI.jpg]

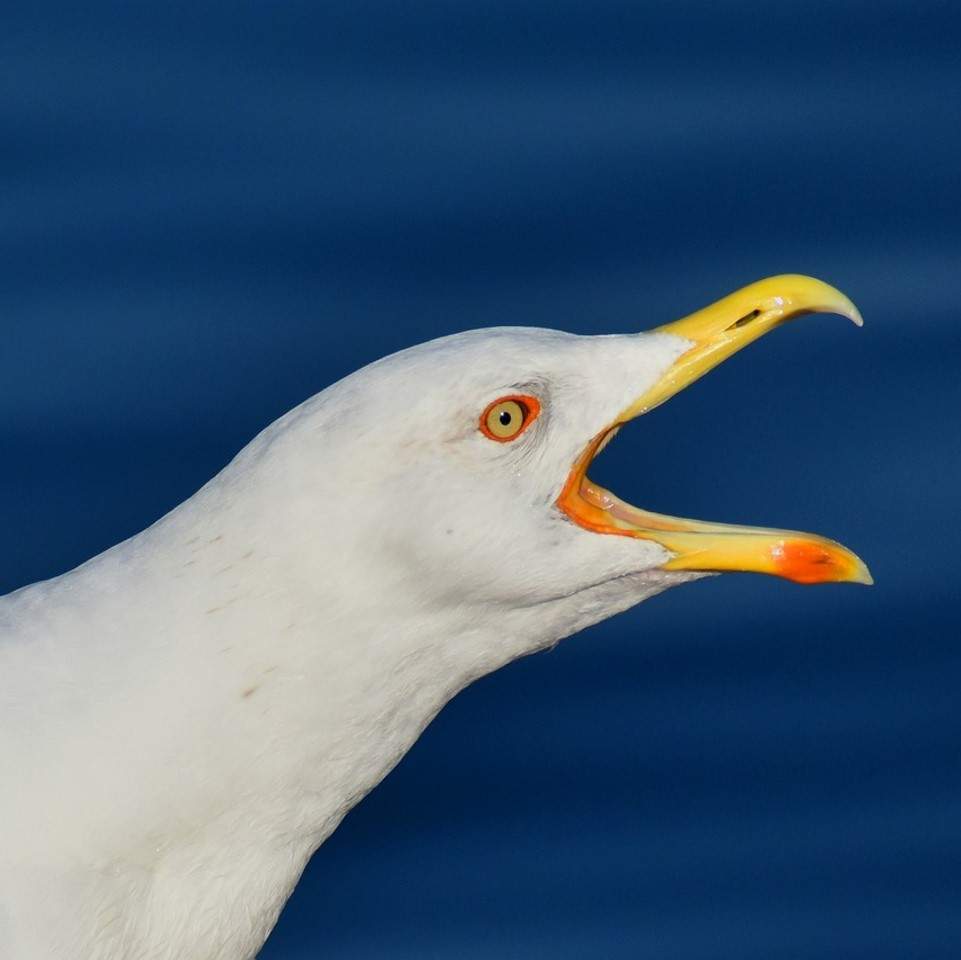

Supplement: Supplementary file 1 — Module Instructions.docxEquitable Care in Telemedicine folderFacilitator Guide for Alternative Teaching Options.docxInterpreter Room for Improvement Example.mp4Interpreter Better Example.mp4Working With Interpreters in Telehealth.pptxTips for Best Practices With Interpreters Handout.docxPostsurvey.docx [file mep_2374-8265.11367-s001.zip › B. Equitable Care in Telemedicine/content/assets/IslcN9PpmgZXVjG2_hhVwCEC2w4T5mRPb.jpg]

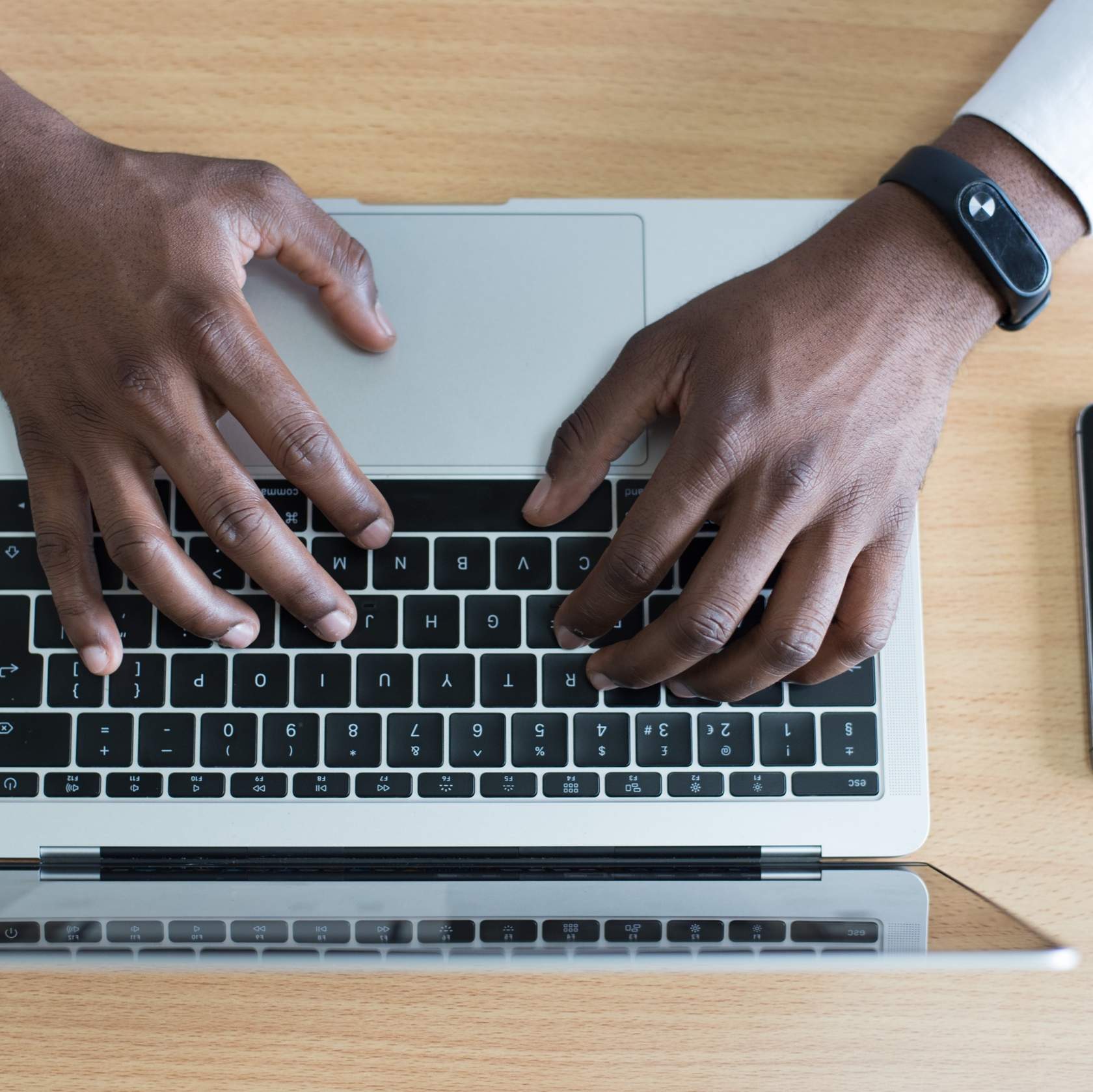

Supplement: Supplementary file 1 — Module Instructions.docxEquitable Care in Telemedicine folderFacilitator Guide for Alternative Teaching Options.docxInterpreter Room for Improvement Example.mp4Interpreter Better Example.mp4Working With Interpreters in Telehealth.pptxTips for Best Practices With Interpreters Handout.docxPostsurvey.docx [file mep_2374-8265.11367-s001.zip › B. Equitable Care in Telemedicine/content/assets/IVa4QPr4UOCpkt_I_6a5Blr9Yg6WqOHi5.jpg]

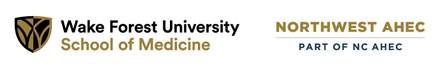

Supplement: Supplementary file 1 — Module Instructions.docxEquitable Care in Telemedicine folderFacilitator Guide for Alternative Teaching Options.docxInterpreter Room for Improvement Example.mp4Interpreter Better Example.mp4Working With Interpreters in Telehealth.pptxTips for Best Practices With Interpreters Handout.docxPostsurvey.docx [file mep_2374-8265.11367-s001.zip › B. Equitable Care in Telemedicine/content/assets/ldmgypWpz8SqEZP6_AssJk6i7KLIVN-5b.jpg]

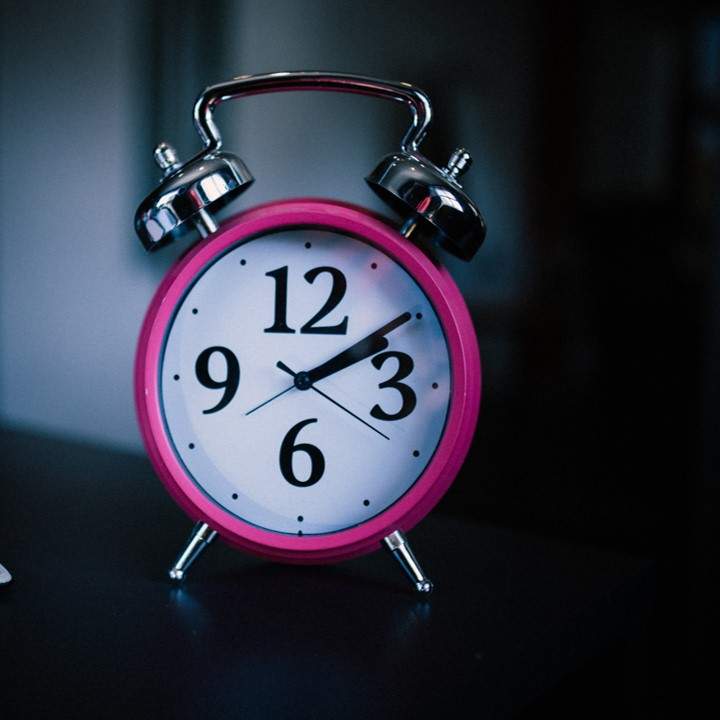

Supplement: Supplementary file 1 — Module Instructions.docxEquitable Care in Telemedicine folderFacilitator Guide for Alternative Teaching Options.docxInterpreter Room for Improvement Example.mp4Interpreter Better Example.mp4Working With Interpreters in Telehealth.pptxTips for Best Practices With Interpreters Handout.docxPostsurvey.docx [file mep_2374-8265.11367-s001.zip › B. Equitable Care in Telemedicine/content/assets/Mdw6kTPuOcwGj8c2_Krw06Qlp5qSqK5LM.jpg]

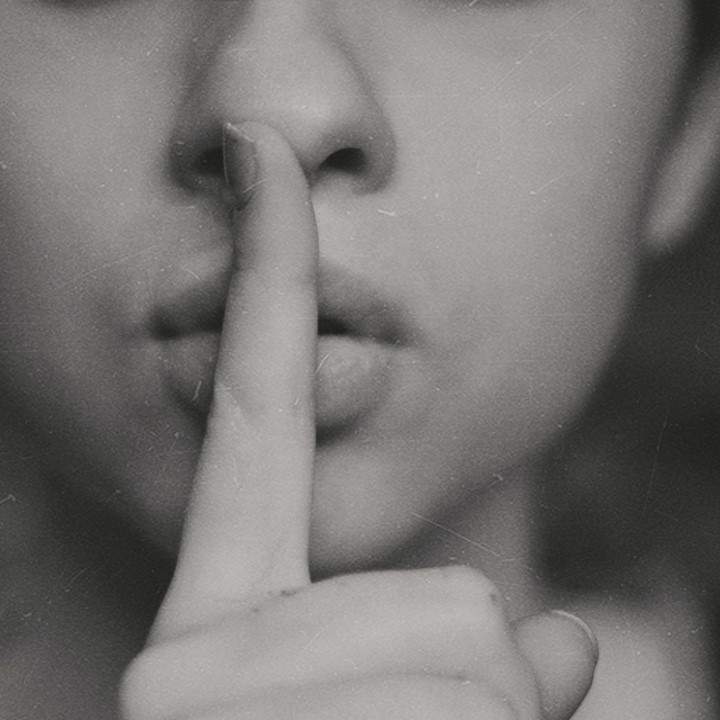

Supplement: Supplementary file 1 — Module Instructions.docxEquitable Care in Telemedicine folderFacilitator Guide for Alternative Teaching Options.docxInterpreter Room for Improvement Example.mp4Interpreter Better Example.mp4Working With Interpreters in Telehealth.pptxTips for Best Practices With Interpreters Handout.docxPostsurvey.docx [file mep_2374-8265.11367-s001.zip › B. Equitable Care in Telemedicine/content/assets/n_Mo0AAaGHGrW_9j_qMJYNeXzQAPZDvZZ.jpg]

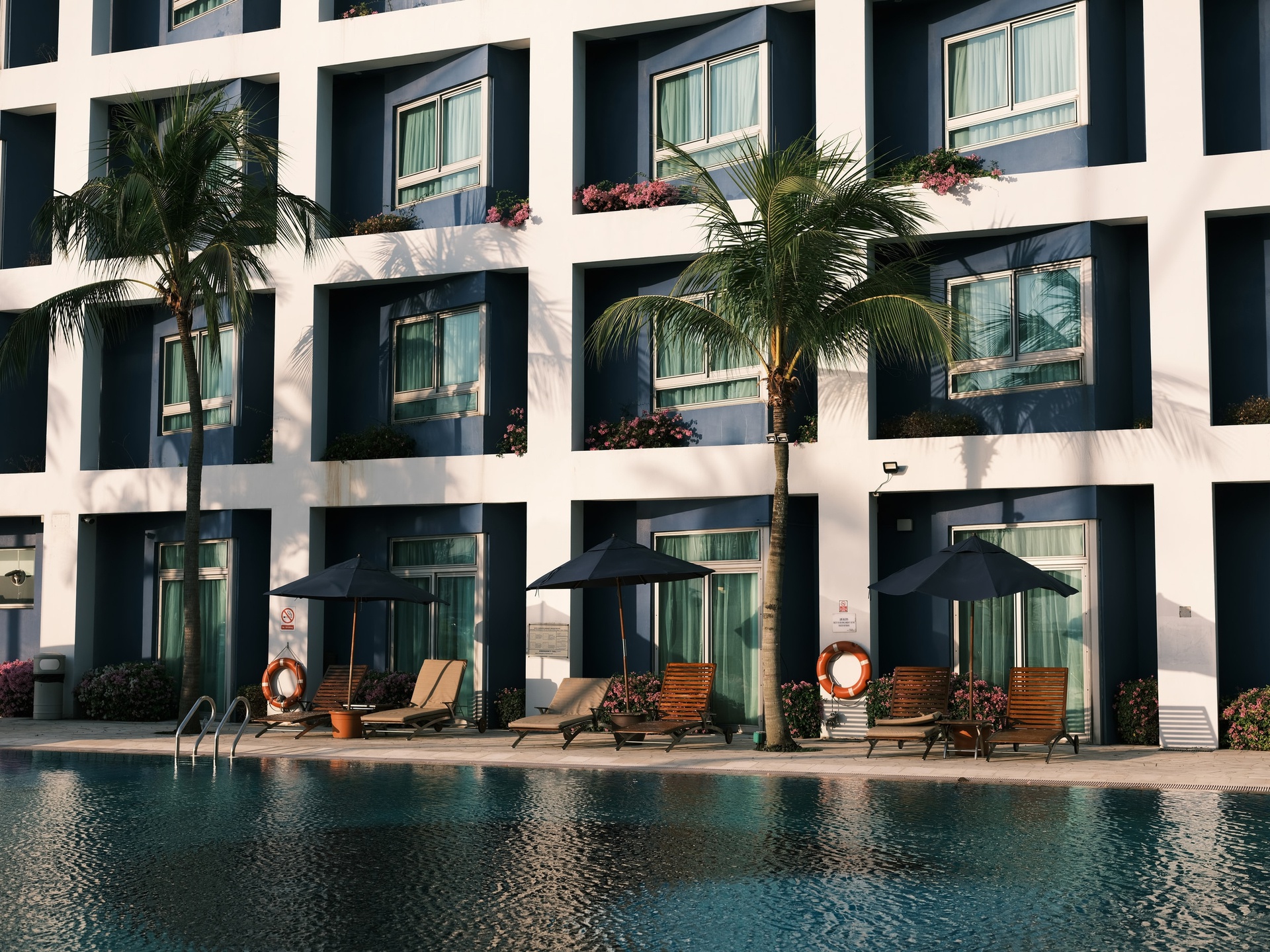

Supplement: Supplementary file 1 — Module Instructions.docxEquitable Care in Telemedicine folderFacilitator Guide for Alternative Teaching Options.docxInterpreter Room for Improvement Example.mp4Interpreter Better Example.mp4Working With Interpreters in Telehealth.pptxTips for Best Practices With Interpreters Handout.docxPostsurvey.docx [file mep_2374-8265.11367-s001.zip › B. Equitable Care in Telemedicine/content/assets/TQjd-2ntFL_THoVQ_13_hospitality.jpg]

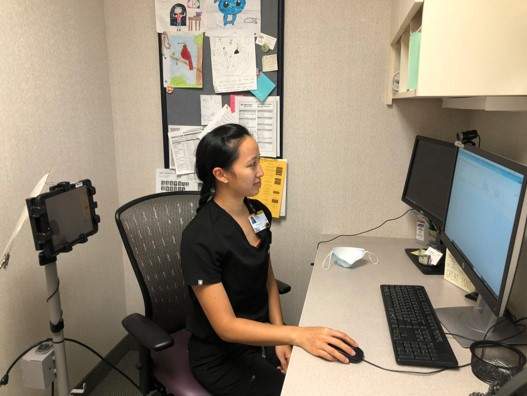

Supplement: Supplementary file 1 — Module Instructions.docxEquitable Care in Telemedicine folderFacilitator Guide for Alternative Teaching Options.docxInterpreter Room for Improvement Example.mp4Interpreter Better Example.mp4Working With Interpreters in Telehealth.pptxTips for Best Practices With Interpreters Handout.docxPostsurvey.docx [file mep_2374-8265.11367-s001.zip › B. Equitable Care in Telemedicine/content/assets/UnUniXfLs_JvTZ2N_kQ4lw3RXak4vU0qu.jpg]

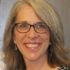

Supplement: Supplementary file 1 — Module Instructions.docxEquitable Care in Telemedicine folderFacilitator Guide for Alternative Teaching Options.docxInterpreter Room for Improvement Example.mp4Interpreter Better Example.mp4Working With Interpreters in Telehealth.pptxTips for Best Practices With Interpreters Handout.docxPostsurvey.docx [file mep_2374-8265.11367-s001.zip › B. Equitable Care in Telemedicine/content/assets/WeEFd8T2Y_Ew_cJ1_small_1672683536.png]

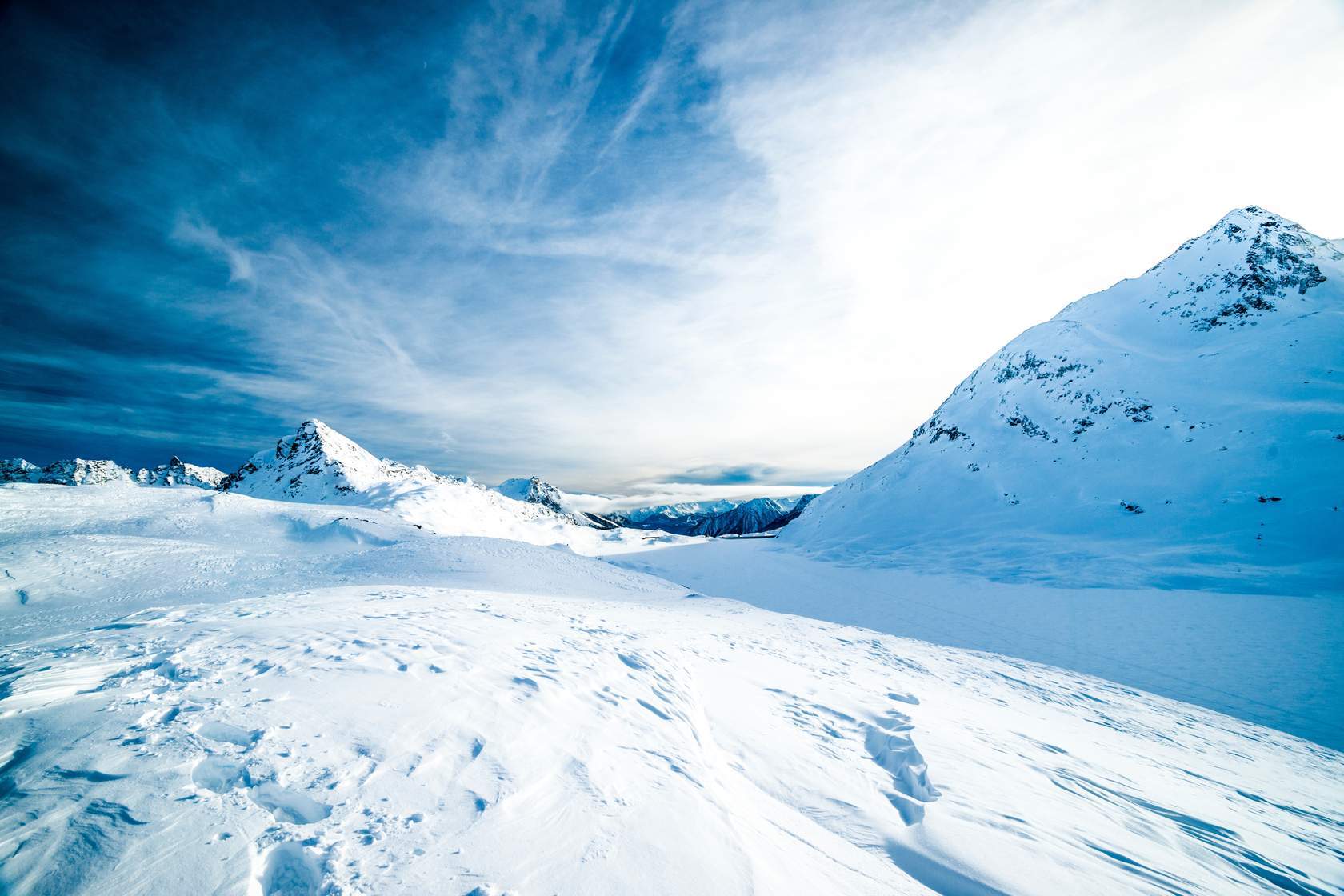

Supplement: Supplementary file 1 — Module Instructions.docxEquitable Care in Telemedicine folderFacilitator Guide for Alternative Teaching Options.docxInterpreter Room for Improvement Example.mp4Interpreter Better Example.mp4Working With Interpreters in Telehealth.pptxTips for Best Practices With Interpreters Handout.docxPostsurvey.docx [file mep_2374-8265.11367-s001.zip › B. Equitable Care in Telemedicine/content/assets/WPuUa6fnKpyEDM1__mountains.jpg]

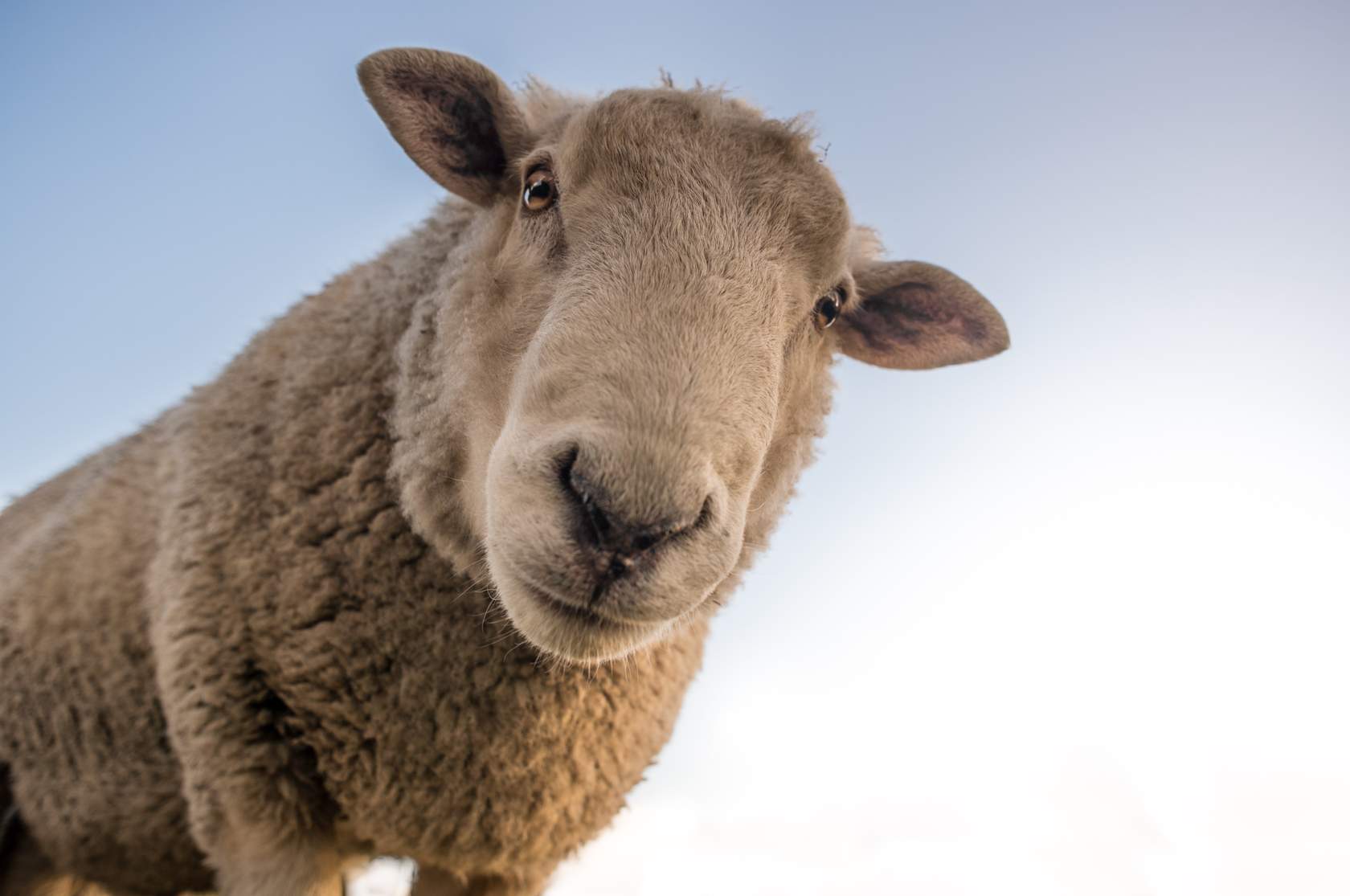

Supplement: Supplementary file 1 — Module Instructions.docxEquitable Care in Telemedicine folderFacilitator Guide for Alternative Teaching Options.docxInterpreter Room for Improvement Example.mp4Interpreter Better Example.mp4Working With Interpreters in Telehealth.pptxTips for Best Practices With Interpreters Handout.docxPostsurvey.docx [file mep_2374-8265.11367-s001.zip › B. Equitable Care in Telemedicine/content/assets/WUQ_edMEfYgURp0B_WdQRLRlBtiDF1Pbe.jpg]

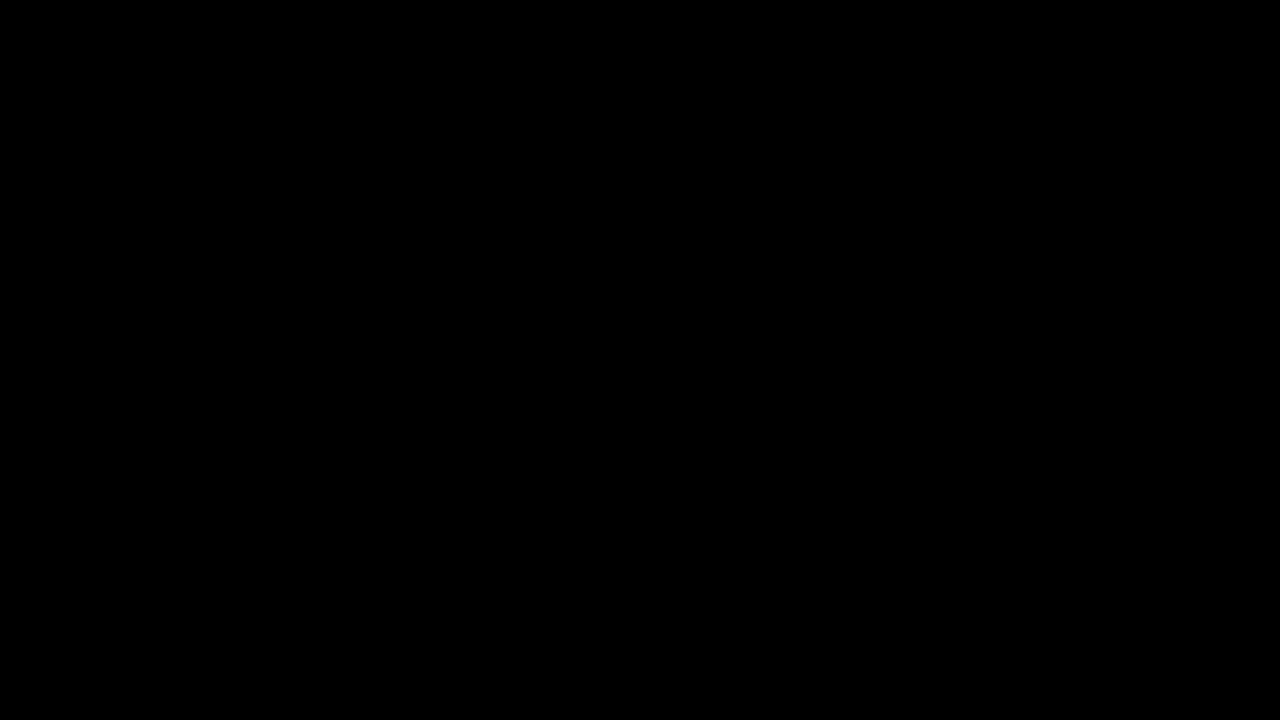

Supplement: Supplementary file 1 — Module Instructions.docxEquitable Care in Telemedicine folderFacilitator Guide for Alternative Teaching Options.docxInterpreter Room for Improvement Example.mp4Interpreter Better Example.mp4Working With Interpreters in Telehealth.pptxTips for Best Practices With Interpreters Handout.docxPostsurvey.docx [file mep_2374-8265.11367-s001.zip › B. Equitable Care in Telemedicine/content/assets/XWAy8ihJRx0q9Wgh_transcoded-a8N_G685GP5KJXqI-telehealth-bad-example-1-00001.png]
